# Design of Novel TRPA1 Agonists Based on Structure of Natural Vasodilator Carvacrol—In Vitro and In Silico Studies

**DOI:** 10.3390/pharmaceutics16070951

**Published:** 2024-07-18

**Authors:** Đorđe Đukanović, Relja Suručić, Milica Gajić Bojić, Saša M. Trailović, Ranko Škrbić, Žarko Gagić

**Affiliations:** 1 Centre for Biomedical Research, Faculty of Medicine, University of Banja Luka, 78000 Banja Luka, Bosnia and Herzegovina; milica.gajic@med.unibl.org (M.G.B.); ranko.skrbic@med.unibl.org (R.Š.); 2 Department of Pharmacy, Faculty of Medicine, University of Banja Luka, 78000 Banja Luka, Bosnia and Herzegovina; relja.surucic@med.unibl.org (R.S.); zarko.gagic@med.unibl.org (Ž.G.); 3 Department of Pharmacology, Toxicology and Clinical Pharmacology, Faculty of Medicine, University of Banja Luka, 78000 Banja Luka, Bosnia and Herzegovina; 4 Department of Pharmacology and Toxicology, Faculty of Veterinary Medicine, University of Belgrade, 11000 Belgrade, Serbia; sasa@vet.bg.ac.rs

**Keywords:** 3D-QSAR, molecular docking, TRPA1, carvacrol, hypertension

## Abstract

Considering the escalating global prevalence and the huge therapeutic demand for the treatment of hypertension, there is a persistent need to identify novel target sites for vasodilator action. This study aimed to investigate the role of TRPA1 channels in carvacrol-induced vasodilation and to design novel compounds based on carvacrol structure with improved activities. In an isolated tissue bath experiment, it was shown that 1 µM of the selective TRPA1 antagonist A967079 significantly (*p* < 0.001) reduced vasodilation induced by 3 mM of carvacrol. A reliable 3D-QSAR model with good statistical parameters was created (R^2^ = 0.83; Q^2^ = 0.59 and $R_{pred}^{2}$ = 0.84) using 29 TRPA1 agonists. Obtained results from this model were used for the design of novel TRPA1 activators, and to predict their activity against TRPA1. Predicted pEC50 activities of these molecules range between 4.996 to 5.235 compared to experimental pEC50 of 4.77 for carvacrol. Molecular docking studies showed that designed molecules interact with similar amino acid residues of the TRPA1 channel as carvacrol, with eight compounds showing lower binding energies. In conclusion, carvacrol-induced vasodilation is partly mediated by the activation of TRPA1 channels. Combining different in silico approaches pointed out that the molecule D27 (2-[2-(hydroxymethyl)-4-methylphenyl]acetamide) is the best candidate for further synthesis and experimental evaluation in in vitro conditions.

## 1. Introduction

The prevalence of hypertension in adults has increased worldwide in the last three decades, especially among the population of low- and middle-income countries. Affecting approximately 1.3 billion people in the world, hypertension still remains undiagnosed in nearly 50% of adults who suffer from this condition [1]. Hypertension can also evoke other health complications such as stroke, heart attack, and kidney dysfunction [2]. Another challenging condition is prehypertension. Although prehypertension does not demand conventional therapy, most patients do not comply with the hygienic–dietary recommendations, which finally results in the development of hypertension [3]. In addition, control of gestational hypertension was also proved to be crucial in pregnant women admitted with preeclampsia [4]. Commonly, the onset of these conditions is associated with the changes in regulation of vascular tone and vasodilators are widely used drugs in the treatment of elevated blood pressure. Considering previously described health issues there is an ongoing necessity to identify a new target site for vasodilator action as well as to discover novel molecules with the vasorelaxant effect.

Members of *Lamiaceae* family, which include plants such as oregano, are traditionally used in the treatment of hypertension [5]. Extracts from the oregano plant showed a vasorelaxant effect in vitro [6]. The main compound in these extracts responsible for their biological effects is the monoterpene carvacrol, which is abundantly found in essential oils extracted from plants belonging to the *Lamiaceae* family [7,8]. Essential oils rich in monoterpenes have been reported to have vasorelaxant properties, potentially explaining their traditional use in treating hypertension [9]. With its anti-inflammatory, antioxidant, and vasorelaxant effects, carvacrol shows significant potential to be used in the treatment of cardiovascular diseases such as hypertension. Carvacrol decreased the values of blood pressure in anesthetized normotensive rats when administered intraperitonealy [10]. Indeed, oral treatment with carvacrol also showed a hypotensive effect in spontaneously hypertensive rats, simultaneously causing a reduction in peripheral vascular resistance [11,12]. Although the exact mechanism of carvacrol-induced vasodilation has not been clarified, it is reported that its action involves activation and/or blockade of several ion channels including transient receptor potential (TRP) channels [13].

TRP channels enable the transport of cations through the cell membrane, thus regulating different physiological processes [14]. Among six TRP subfamilies, TRP ankyrin (TRPA) channels represent the smallest subfamily consisting of only one member [15]. TRPA1 channels were first found in sensory neurons where they take part as important ion channels in pathophysiological processes such as pain, itch, and inflammation [16]. In the following years, it was revealed that TRPA1 channels are significantly involved in both physiological and pathophysiological processes in cardiovascular regulation. Recent reports showed that TRPA1 channels are also present in cardiomyocytes and endothelial cells of blood vessels [17,18]. Considering their presence and involvement in cardiovascular functions, TRPA1 channels could be considered a promising target for the development of treatments for several cardiovascular diseases with a focus on heart failure, atherosclerosis, myocardial fibrosis, myocardial ischemia-reperfusion injury, and hypertension [19,20,21]. Among numerous compounds that can activate these channels, natural molecules such as carvacrol, thymol, cinnamaldehyde, and similar stand out as very potent TRPA1 agonists [22,23].

Therefore, one of the aims of this study was to investigate the role of TRPA1 channels in carvacrol-induced vasodilation. Additionally, the direction of this research is intended to describe interactions between carvacrol and the target site of TRPA1 channels and to use those data to design new molecules with greater activity on these channels.

## 2. Materials and Methods

### 2.1. In Vitro Experiments

#### 2.1.1. Drugs and Solutions

Potassium chloride (KCl), sodium chloride (NaCl), magnesium sulfate (MgSO_4_), calcium chloride (CaCl_2_), glucose (C_6_H_12_O_6_), potassium phosphate (KH_2_PO_4_), sodium carbonate (NaHCO_3_), barium chloride (BaCl_2_), and ethylenediaminetetraacetic acid (EDTA) were obtained from Lach-Ner s.r.o. (Neratovice, Czech Republic) and used to prepare Krebs–Ringer bicarbonate solution and Ca^2+^—free Krebs–Ringer bicarbonate solution. Other substances included phenylephrine (PE; purity ≥ 98%), carvacrol (purity ≥ 98%), isopentenyl pyrophosphate (IPP; purity ≥ 95%), and A967079 were purchased from Sigma Aldrich (St. Louis, MO, USA). Stock solutions were prepared either in distilled water or in 99.9% ethanol, according to the solubility of substances. Consecutive dilutions were carried out in distilled water and stored at 0–4 °C.

#### 2.1.2. Tissue Preparation 

After surgical procedures at the Clinics for Abdominal Surgery, University Clinical Centre of the Republic of Srpska, Banja Luka the samples of the subsegment branch of the superior human mesenteric artery (HMA) were placed into the container filled with cooled modified Krebs-Ringer bicarbonate solution and transported by cold chain to the Centre for Biomedical Research, Faculty of Medicine, University of Banja Luka. Samples were used for experiments immediately after they were received in the laboratory. The mesenteric arteries were isolated and cleaned from surrounding connective tissue, after which they were cut into rings 3–4 mm in length. These procedures were performed in Petri dishes placed on flat ice packs. HMA rings, after two stainless steel hooks were pulled through their lumen, were suspended in a glass chamber filled with 10 mL of Krebs-Ringer bicarbonate solution (37 °C; pH 7.4), aerated with a mixture of 95% O_2_ and 5% CO_2_. One hook was fastened to the transducer (MDE Research, Budapest, Hungary) that is connected to the amplifier, allowing the recording of changes in isometric tension using isoSys SOFT-02 software. Simultaneously, another hook was attached to the displacement unit, which allowed fine adjustment of resting tension.

#### 2.1.3. Experimental Protocols

In order to obtain optimal results, each HMA ring was exposed to experimental conditions formerly defined in our laboratory [24,25]. After a 30 min adaptation period, preparations were gently adjusted to the passive tension of 2 g and incubated for 120 min. During this time, 40 mmol/L KCl was administrated three times (30, 90, and 120 min). Between each administration medium was changed (every 10 min) and passive tension was set again at 2 g. Firstly, the vasorelaxant effect of carvacrol in isolated HMA was obtained by creating a concentration–response curve. Rings were pre-contracted with 1 μmol/L PE and when contraction reached a stable plateau, increasing concentrations of carvacrol (1–3000 μmol/L) were added. To investigate the role of TRPA1 channels in vasodilation of HMA, the rings were incubated with 10 μmol/L IPP (non-selective TRPA1 antagonist) or 1 μmol/L A967079 (selective TRPA1 antagonist) for 30 min and then carvacrol concentration–response curves were obtained (Figure S1) [26,27]. Vasorelaxant responses were presented as a percentage of decline in PE-induced pre-contraction and concentration–response curves were compared with representative control curves.

#### 2.1.4. Ethical Approval

Sample collection and experiments were approved by the Ethics Committee of the University Clinical Centre of the Republic of Srpska, Banja Luka, the Republic of Srpska, Bosnia and Herzegovina (No. 01-19-491-2/23).

#### 2.1.5. Statistical Analysis

The statistical analysis was performed in SigmPlot version 14.0 software, while graphical representation was created in GraphPad Prism 6.0 software. Two-way ANOVA, followed by the Bonferroni test, was performed. The results were expressed as mean ± standard error of the mean (SEM) and were considered statistically significant if they had a null hypothesis probability of less than 5% (*p* < 0.05).

### 2.2. In Silico Experiments

#### 2.2.1. 3D-QSAR 

The dataset was created by obtaining small molecules that activate TRPA1 channels from the ChEMBL database. Negative logarithm values (pEC_50_) for EC_50_ activities that were previously converted to molar concentration were calculated. Activities of all compounds were measured under the same experimental condition. The dataset was divided into two groups, one with 20 compounds representing the training set used for partial least square (PLS) analysis and another with 9 compounds representing the test set used for external validation (Table S1). The determination of pKa values and the selection of dominant molecular forms at pH 7.4 were conducted using MarvinSketch 5.5.1.0 software [28]. Dominant structures were then optimized with the Hartree–Fock method using the Gaussian software included in the Chem3D Ultra 7.0 program [29]. 

The 3D quantitative structure–activity relationship (3D-QSAR) of TRPA1 agonists was investigated with Pentacle 1.07 software [30], which uses GRIND (GRid INdependent Descriptors) obtained from molecular interaction fields [31]. For the calculation of molecular interaction fields, 4 different probes were used: DRY (represents hydrophobic interactions), O (sp2 carbonyl oxygen, representing H-bond acceptor groups), N1 (amide nitrogen, representing H-bond donor groups), and the TIP probe (representing steric hot spots). The ALMOND algorithm was employed to identify and select 100 regions (nodes) with the most favorable interactions for each probe. Nodes were transformed to GRIND using the consistently large auto and cross-correlation (CLACC) algorithm and the values were presented in the form of a correlogram where each peak represents one variable. Selection of the most useful variables was done using fractional factorial design (FFD). The partial least squares (PLS) regression method was used to analyze the relationship between obtained variables and activities. In order to determine the statistical quality of created 3D-QSAR (TRPA1) models, their internal and external validation parameters were calculated. Internal validation represents the calculations performed only on molecules from the training set and includes the following parameters: squared correlation coefficient or coefficient of determination (R^2^), cross-validated coefficient of determination (Q^2^), and Root Mean Square Error of Estimation (RMSEE). The Q^2^ was calculated using the leave one out (LOO-) approach, where a new model is formed by eliminating each compound from the training set once, after which this model is used to predict the Y-value of the removed compound. The difference in observed and predicted activity (e_(i)_) was calculated for each compound and used to determine PRESS (Predicted Sum of Squares), RMSEE, and Q^2^.

$$PRESS=\sum_{i=1}^{n}e_{\left(i\right)}^{2}$$

$$RMSEE=\sqrt{\frac{PRESS}{n}}$$

$$Q2=1-\frac{PRESS}{\sum {\left(Y_{obs(training)}-{\bar{Y}}_{training}\right)}^{2}}$$

The PLS model can be considered as reliable if: R^2^ > 0.6; Q^2^ > 0.5 and RMSEE < 0.5 [32,33]. As a parameter of external validation, the predictive correlation coefficient ($R_{pred}^{2}$) was calculated using molecules from the test set:

$$R_{pred}^{2}=1-\frac{\sum {\left(Y_{obs(test)}-{\bar{Y}}_{pred(test)}\right)}^{2}}{\sum {\left(Y_{obs(test)}-{\bar{Y}}_{training}\right)}^{2}}$$

Additionally, $r_{m}^{2}$ metrics parameters ($r_{m}^{2}$, $r_{m}^{‘2}$, ${\bar{r}}_{m}^{2}$, ${\Delta r}_{m}^{2}$), that represents the strictest criterion of external validation were also calculated. For the predictive QSAR model, the values of these parameters should be $R_{pred}^{2}$ > 0.5; $r_{m}^{2}$, $r_{m}^{‘2}$ and their mean ${\bar{r}}_{m}^{2}$ > 0.5; while their difference ${\Delta r}_{m}^{2}$ < 0.2 [33,34].

Finally, the applicability domain, which defines the response and chemical structure space in which the QSAR model makes predictions with a given reliability, was determined [35].

#### 2.2.2. ADMET Prediction

One of the aims of drug discovery is to identify the drug candidates possessing favorable ADMET properties, thus minimizing the risk of obtaining inadequate compounds during the early stages of drug development. In this study, the ADMET Predictor (TM) version 11.0, 64-bit edition (accessed by cloud) was used to predict various ADMET parameters related to carvacrol and designed compounds [36].

#### 2.2.3. Molecular Docking

The cryo-EM structure of TRPA1 (PDB: 6X2J) in complex with ligand was obtained from Protein Data Bank (PDB) and used for AutoDock Vina docking protocol [37,38]. Prior to the beginning of the docking protocol, both ligand and receptor needed to be prepared using AutoDockTools 1.5.6 [39]. Polar hydrogens and charges were added to the ligand as well as to the receptor, and then water molecules were removed from the receptor and torsions were added to the ligand. The co-crystallized ligand located at the center of the grid box was used to create the docking grid with a grid point spaced at 1 Å. The acquired coordinates were saved in the configuring file and used in the docking process of tested molecules. The validation parameter for molecular docking is root mean square deviation (RMSD) and it was calculated by comparing the docked with the co-crystallized pose of the ligand using Discovery Studio Visualizer v4.1 [40]. The value of this parameter should be as near as possible to 0, while the model is considered reliable if RMSD < 2 [41].

#### 2.2.4. Molecular Dynamic Simulation

The molecular dynamic (MD) simulation was performed for the two most stable complexes: 6X2J-carvacrol and 6X2J-most promising designed TRPA1 agonist, using YASARA Structure v.20.12.24.2.64 software. The experimental setup included hydrogen-bond optimization and pKa prediction for the selected pH value (7.4) [42]. The simulation was run for 50 ns with the AMBER 14 force field, while the setup conditions for temperature and pressure were 298 K and one atmosphere, respectively. Three validation parameters were analyzed: total potential energy of the simulated systems, radius of gyration (Rg), and RMSD of ligand conformation changes during the simulation.

## 3. Results and Discussion

### 3.1. Organ Bath

**Figure 1:**
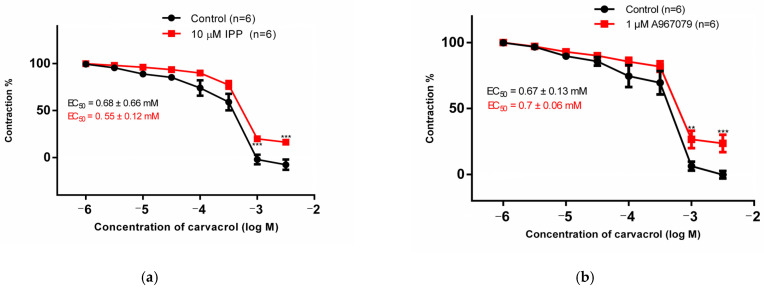
The graph shows the relaxant effect of carvacrol (CRV; 1–3000 µM) on HMA pre-incubated with (**a**) IPP (10 µM) or (**b**) A967079 (1 µM) and then pre-contracted with 1 µM PE. Values are expressed as percentage of primary PE contraction, which is referred to as 100% (mean ± SEM); (** *p* < 0.01; *** *p* < 0.001; two-way ANOVA, followed by Bonferroni test). The EC_50_ of the vasorelaxant effect of carvacrol was calculated for each curve, both in the presence and absence of TRPA1 antagonists.

Organ bath methodology was used to evaluate the role of TRPA1 channels in carvacrol-induced vasodilation. After pre-incubation of HMA rings with non-selective TRPA1 antagonist (10 µM IPP), the maximum vasorelaxant effect caused by 3 mM of carvacrol was decreased from 107.6% to 83.7% (Figure 1a). Similar results were observed when the preparations were incubated with TRPA1 selective blocker A967079 (Figure 1b). The efficacy of carvacrol was significantly reduced in the presence of A967079 or IPP, whereas its potency showed little to no change, as indicated by minimal changes in the EC50 values. Control vehicle curves are not shown in the graph, but they were stable over time. 

Although the maximal effect of carvacrol-induced vasodilation was reduced in presence of the either IPP or A967079, carvacrol still caused vasorelaxation of pre-contracted HMA at high concentrations. This indicates the possible involvement of other mechanisms besides the activation of TRPA1 channels in carvacrol-induced vasodilation.

As a constituent of essential oil, the ability of carvacrol to dilate smooth muscles was first described on isolated rat uteri [43]. Several studies confirmed the vasorelaxant effect of carvacrol even in human blood vessels [13]. Although carvacrol can cause hypotensive effects by inducing vasodilation, the exact mechanism of its action is still not completely explained. The blocking of L-type voltage-gated Ca^2+^ channels was most often proposed as a leading mechanism of the vasorelaxant effect caused by monoterpenoid compounds [13,44,45]. Both carvacrol in the concentration of 3 mM and similar monoterpene carveol in the concentration of 5 mM managed to relax pre-contracted isolated human umbilical arteries regarding the above-mentioned mechanism [13,45]. Testai et al. reported that 3 mM of 4 aminopyridine and 200 μM of quinine (blockers of voltage-gated potassium channels; K_v_) reduced the vasodilation caused by lower concentrations of carvacrol (0.1 to 50 μM) in rat aorta, indicating the involvement of K_v_ channels in the mechanism of its action [46]. Carvacrol is also a well-known agonist of TRPA1, channels whose activation can engage further mechanisms, resulting in the relaxation of blood vessels. In recent years, there have been numerous studies investigating the role of TRP channels in the regulation of vascular tone, with emphasis on the TRPA1 channel. Bautista et al. reported that activation of the TRPA1 channel on perivascular nerve endings can cause vasodilation of isolated rat mesenteric arteries [47]. Several articles showed that formaldehyde, zinc pyrithione, and α-pinene also caused vasorelaxation of the murine mesenteric artery including TRPA1 activation as a leading mechanism [48,49,50,51]. Activation of TRPA1 channels caused either neurogenic vasodilation or endothelial-dependent vasodilation of isolated animal arteries [52,53]. The increase in dermal blood flow evoked by topically applied cinnamaldehyde (TRPA1 agonist) on the human forearm was diminished by TRPA1 antagonist LY3526318 [53]. An intravenously injected lower dose of cinnamaldehyde caused a significant hypotensive effect in wild-type mice compared to TRPA1 knockout mice [54]. In light of the aforementioned studies and our results, it is evident that these channels are integral to the processes that regulate vascular tone.

### 3.2. 3D-QSAR

In silico methods were applied in order to design novel molecules based on carvacrol structure with better activity on TRPA1 channels which could result in possibly better vasorelaxant effect. Alongside other in silico models, a credible QSAR model was developed to identify and predict the inhibitory effects on TRPA1 channels among known approved substances [55]. As far as we know, no QSAR model has been developed to predict agonistic activity on TRPA1 channels.

A reliable 3D-QSAR model for the prediction of TRPA1 agonist activities was created using molecules from the training set. Initially, the entire set of GRIND variables was used to build the PLS model in order to select a significant pharmacophore. To obtain a non-over-fitted model, the number of variables was reduced using the FFD selection algorithm. As can be seen from Table 1, values of both internal validation (R^2^ = 0.83 and Q^2^ = 0.59) and external validation parameters ($R_{pred}^{2}$ = 0.84216; $r_{m}^{2}$ = 0.75732; ${\bar{r}}_{m}^{2}$ = 0.61483; $r_{m}^{‘2}$ = 0.68608; ${\Delta r}_{m}^{2}$ = 0.14249) indicate the good predictive ability of the 3D-QSAR model. Additionally, the determined applicability domain found no outliers in the dataset.

**Figure 2:**
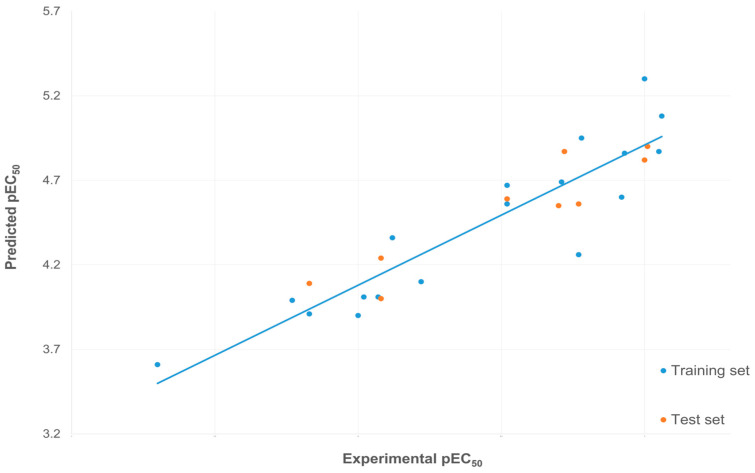
The plot of experimental and predicted TRPA1 activators activities expressed as pEC_50_.

The model’s good predictive power could be also observed in the plot of experimental versus predicted values, where the even distribution of pEC_50_ values around the regression line indicates slight deviations between experimental and predicted activities for all compounds in the dataset (Figure 2). It is important to note that the range of experimental pEC50 values observed for the L compound is narrow. However, this range of pEC50 values is typical among small molecules known to activate TRPA1, such as carvacrol.

The most important variables in the created PLS model were selected to explain the impact of present structural characteristics on TRPV1 activity (Table 2). 

**Figure 3:**
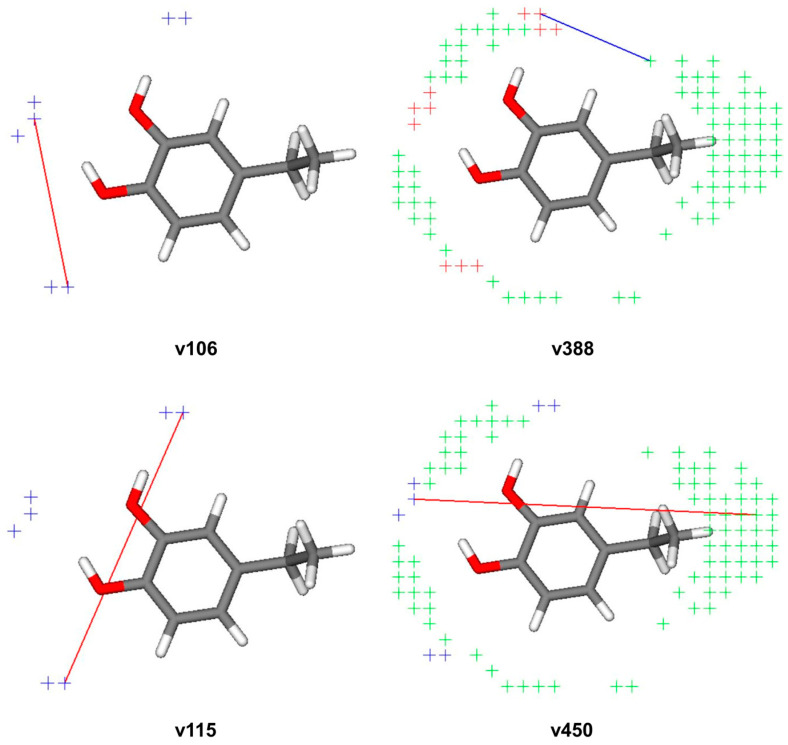
Illustration of the most important variables and their positive and negative influences on the activity of compound L17 against TRPA1. TIP probes are presented in green, O probes in red, and N1 probes in blue. Favorable interactions are depicted with red lines, while unfavorable interactions are presented with blue lines.

Positive and negative influences of pharmacophoric features present in selected compounds on TRPA1 activity are illustrated in Figure 3. The phenyl group created a favorable interaction with the steric region when they were in a meta position on the benzene ring (v450; N1-TIP). However, this same region of the molecule could also result in the most influential negative interaction, when it is presented to a hydrogen bond acceptor (v388; O-TIP). Additionally, when two phenyl groups were placed close one to another on a benzene ring, they formed positive interactions (v106 and v115; N1-N1).

**Figure 4:**
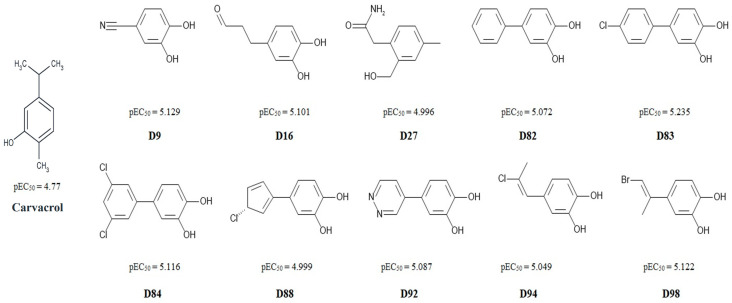
Selection of designed compounds with the highest 3D-QSAR predicted pEC50 values, along with carvacrol and its experimental pEC50 value.

Carvacrol was a chosen compound, which underwent a modification process in order to create novel molecules with an intensified ability to activate TRPA1 channels (Table S2). Described interactions were used as guidelines to modify the structure of carvacrol, which resulted in the design of 10 compounds with enhanced pEC50 values (Figure 4). After designing the structures, we searched the ChEMBL database and found that molecules D9, D82, D83, and D84 were already documented. Despite being known, their activity on TRPA1 channels had not been investigated previously.

Considering that the chemical structure of carvacrol is quite simple, minor changes could create new compounds with significantly altered activities on TRPA1 channels. The replacement of the methyl group in the carvacrol structure with the hydroxyl group proved to be an important adaptation in almost all compounds with enhanced activity. In position 4C on the benzene ring, different structural features showed contribution to the activity such as benzene; 1,3-dichlorobenzene; chlorobenzene; 1-chlorocyclopentane-2,4-dien; pyridazine; propanal; 1-bromoprop-1-en-2-yl; 1-chloroprop-1-en-1-yl. These groups represent steric regions at an optimum distance that is necessary to achieve interactions.

The predicted pEC50 values for D compounds were higher compared to the experimental pEC50 value for carvacrol (4.77) and slightly higher than the pEC50 values of some other compounds used to create the model. Although the validation of the model was successful, providing a reliable tool for predicting TRPA1 pEC50 values, further studies are necessary to determine the EC50 of this compound under experimental in vitro conditions. Compound D27 also showed improved activity although its structure is slightly different from other designed molecules. With the exception of D27, all other D compounds are catechols. The presence of two hydroxyl groups could result in the formation of intramolecular hydrogen bonds. However, strong hydrogen bond interactions between hydroxyl groups in catechols are maintained in weakly interacting solvents, while in strongly interacting solvents such as water, internal hydrogen bonds are lost [56]. As experimentally confirmed by Callear et al., the hydrogens on the hydroxyl groups in dopamine are rather oriented to allow intermolecular hydrogen bonding instead of forming intramolecular hydrogen bonds in catechols [57]. Compound D16 contains an aldehyde group, while compounds D94 and D98 feature alkenes. Both functional groups are highly reactive and could potentially bind covalently to the amino acid residues of TRPA1, particularly cysteine. making them less favorable candidates for synthesis. This risk should be taken into account and evaluated in further in vitro studies.

Considering that all D compounds are small molecules, they may exhibit promiscuity and interact with other receptors. Additionally, TRP channels share structural similarities that could facilitate such interactions. Carvacrol itself, besides activating TRPA1, can also activate TRPV3 channels [58]. Therefore, it could be interesting to evaluate the activity of these compounds on other TRP channels in future studies.

### 3.3. ADMET Properties

The physicochemical and biological properties of selected designed compounds were further estimated in order to eliminate molecules with potentially inadequate ADMET characteristics in the early stages of drug development. Although all compounds showed optimal ADMET properties, only six molecules had improved ADMET scores compared to carvacrol (Table 3). Among those molecules, compound D27 stands out with the lowest ADMET risk, equal to 0. Here, it is also necessary to mention that this model is limited to predicting only CYP metabolism. Considering that almost all D compounds are catechols, these compounds can be substrates for COMT and have shorter durations of action. Compound D27, which showed the most promising predicted ADMET properties, is not catechol and thus cannot be a candidate for COMT metabolism. However, further in vitro and in vivo studies are necessary to precisely evaluate the complete ADMET characteristics of these compounds.

Accurate estimation of synthetic difficulty, whether conducted conceptually or through in silico methods, is essential for prioritizing molecules for synthesis and testing during the early stages of drug discovery. Utilizing an in silico model for this purpose significantly increases the number of synthesis candidates that can be considered. The predicted SynthDiff score for all compounds is low, indicating synthetic accessibility. Alongside carvacrol, compounds D27 and D83 have the lowest scores of 1.52 and 1.82, respectively. In comparison with ADMET Predictor examples benzene (SynthDiff = 0) and riboflavin (SynthDiff = 4), it can be said that the routes to synthesize these molecules should be quite simple. Factors such as a chemist’s creativity, expertise in synthetic chemistry, access to starting materials, and the laboratory equipment at their disposal can all significantly influence the synthesis procedure.

### 3.4. Molecular Docking

While the purpose of creating a 3D-QSAR model was to predict the activities of designed compounds, the molecular docking study allowed us to gain deeper knowledge about binding affinity and interactions between molecules and active sites on the TRPA1 channel. The TRPA1 channel consists of four identical subunits arranged in a tetramer, where each subunit encloses six transmembrane helices [59]. A binding pocket is formed between the first four transmembrane helices (S1–S4) of one subunit and two transmembrane helices (S5-S6) of the neighboring subunit [60]. Considering that carvacrol is a non-covalent activator of TRPA1 channels, a 3D-TRPA1 structure with a co-crystal non-covalent binding ligand (PDB code: 6X2J) was used to create the docking model [60]. The structure of TRPA1 and its cocrystallized ligand were prepared prior to the calculation of the grid box based on the position of the ligand. Docking of selected designed molecules was performed using obtained coordinates from the grid box. The reliability of the docking study was confirmed by calculating root mean square deviation (RMSD). To calculate RMSD, the docked ligand pose should match the determined electron microscopy ligand pose, and the RMSD value has to be lower than 2 Å [41]. In our study, the RMSD value was 0,4179 Å, indicating that the created docking model was reliable. The calculated binding energies of docked designed compounds are lower compared to the binding energy of carvacrol (Table 4).

**Figure 5:**
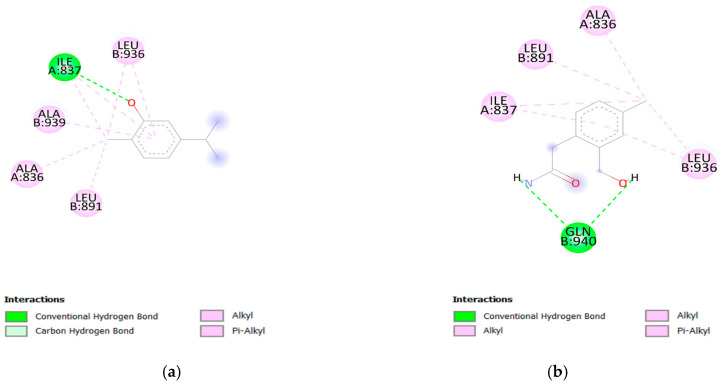
(**a**) Carvacrol and (**b**) Compound D27 docked into TRPA1.

Results showed that 8 out of 10 designed molecules with better-predicted activities also have lower binding energies compared to carvacrol indicating their ability to form a more stable complex with the receptor. By observing carvacrol interactions with TRPA1, it can be seen that residues Ala (A:836), Leu (B891), and Leu (B:936) create alkyl interaction with methyl group, while Leu (B:936) in addition to this alkyl interaction and Ala (B:939) create Pi–alkyl interaction with the benzene ring. Apart from these interactions, Ile (A:837) creates an important hydrogen bond with the phenyl group of carvacrol (Figure 5). The presence of hydroxyl groups, which frequently act as hydrogen bond donors or acceptors, allows designed molecules to interact with residues in the binding pocket of the receptor via hydrogen bonds. Although molecules D82 and D84 had lower binding energies compared with carvacrol, they did not form any hydrogen bonds, which is necessary to ensure better ligand–receptor complex stability. Residues that interact with compound D27 correspond to the residues that interact with carvacrol (Ala (A:836), Leu (B891), Leu (B:936), and Ile (A:837)).

### 3.5. Molecular Dynamic Simulation

In order to further evaluate the stability of carvacrol–TRPA1 and D27–TRPA1 complexes, MD simulation was performed. Evaluating total potential energy, radius of gyration, and RMSD during MD simulations is crucial for understanding the stability and behavior of molecular systems [61]. Each of these parameters offers unique insights into the dynamics and potential effectiveness of molecules, particularly in drug development and biochemical research.

**Figure 6:**
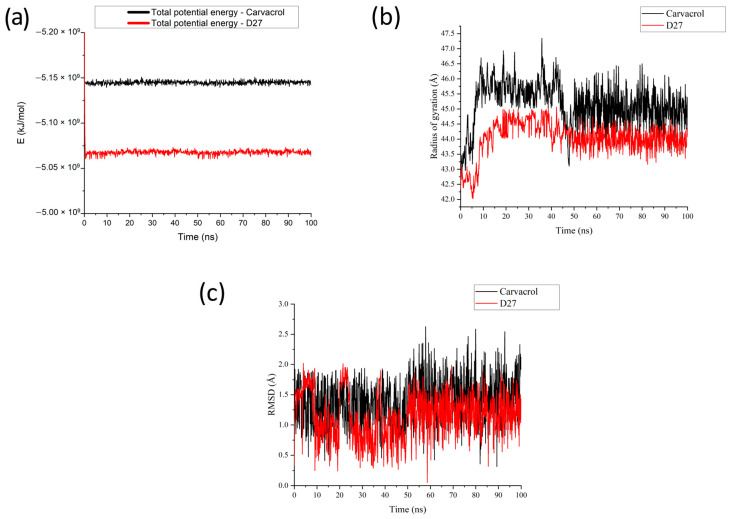
(**a**) Total potential energy (**b**) the radius of the gyration and (**c**) RMSD trajectories of TRPA1 in complexes with carvacrol and compound D27.

Total potential energy is a fundamental measure in MD simulations that reflects the sum of all interatomic forces, including bonds, angles, dihedrals, and non-bonded interactions within a system. A stable MD simulation typically shows the potential energy reaching a plateau or exhibiting minimal fluctuations around a baseline value. This suggests that the molecular system has reached equilibrium, an essential state for accurate biophysical analyses. Changes in the potential energy when comparing derivatives or interactions with different ligands can indicate how modifications affect the stability and reactivity of a molecule. Both carvacrol and D27 appear to reach a stable state relatively quickly as evident from the flattening of the total potential energy curves (Figure 6a).

Carvacrol starts and remains at a lower total potential energy (around −5.15 × 10^9^ kJ/mol) throughout the simulation. This indicates that carvacrol is in a more stable configuration compared to D27 which fluctuates around −5.05 × 10^9^ kJ/mol. Although these fluctuations are minor, they are more pronounced than in carvacrol, which suggests slightly less stability or more dynamic structural adjustments during the simulation. Since carvacrol is a known agonist of the human TRPA1 receptor, the lower and more stable energy profile suggests that its interaction with the receptor might be energetically more favorable [62]. D27, despite being derivative, shows higher energy values, which could imply less favorable interactions or a different mechanism of action, though it still maintains substantial stability. Since D27 is a structural modification of carvacrol aimed at enhancing certain properties (such as solubility, receptor affinity, or specificity), its higher energy state might be a trade-off for other beneficial properties. These energy profiles could guide further modifications to optimize D27’s interaction with TRPA1 or other targets.

The carvacrol–TRPA1 complex shows a higher radius of gyration overall, indicating that it is less compact compared to the D27–TRPA1 complex (Figure 6b). At first, it fluctuated between approximately 43.5 Å to 47.5 Å, additionally stabilizing in the range between 44.5 Å to 44.6 Å in the period from 50 ns to 100 ns of the simulation. The larger fluctuations might suggest that carvacrol in complex undergoes more conformational changes during this period of the simulation. However, D27 maintains a consistently lower radius of gyration, first ranging around 42.5 Å to 45 Å, and then from 50 ns to 100 ns ranging between 43.5 Å to 44.5 Å, suggesting a more stable conformation compared to carvacrol. The greater range and higher value of the radius of gyration might indicate that carvacrol can conform differently in response to environmental changes, potentially allowing for various interactions with the TRPA1 receptor.

Figure 6c shows the RMSD of carvacrol and its derivative D27 over a 100 ns molecular dynamics simulation. RMSD is a common measure used to assess the conformational stability of a molecule relative to a reference structure across the simulation time. Carvacrol shows RMSD values fluctuating broadly between approximately 0.5 Å and 2.25 Å, similar to those observed for D27, indicating structural stability of the analyzed complexes during the MD simulation. These curves suggest that both ligands, once they adopt a conformation compatible with their target, can maintain this interaction with greater consistency.

**Figure 7:**
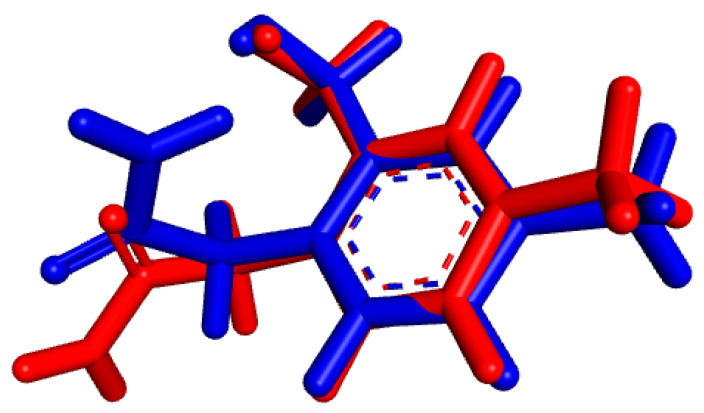
Blue represents the conformation of compound D27 from docking studies, while red indicates the conformation of compound 27 with minimum energy observed during the MD simulation.

Previous docking simulation studies have proposed an optimal conformation for interaction with the receptor. We compared this conformation to the one with minimum energy observed during the simulation, as illustrated in Figure 7. The calculated RMSD between these conformations is 0.3096 Å, confirming the conformational stability of D27 throughout the simulation, which closely mimics physiological conditions

One of the key strengths of this study is the use of human blood vessels in the in vitro experiments, significantly enhancing the relevance and applicability of our findings to human physiology and potential therapeutic interventions. To our knowledge, this study is the first to evaluate the role of TRPA1 channels in human blood vessels. Furthermore, the combination of various in silico approaches provides detailed insights into the properties of the designed molecules.

However, this study also has its limitations. The organ bath setup isolates tissue from its natural environment, which excludes systemic interactions and may affect the accuracy of in vivo replication. Although the validation parameters of the 3D-QSAR model are satisfactory, a more homogeneous dataset with additional similar molecules activating TRPA1 could further enhance modeling accuracy.

Our future research will focus on synthesizing the suggested compounds and experimentally testing their activities and ADMET properties to validate our in silico predictions.

## 4. Conclusions

The present study showed that vasorelaxant effect caused by carvacrol in HMA model was partially reversed by the application IPP (10 µM) and A967079 (1 µM), further indicating the involvement of TRPA1 channels. A reliable 3D-QSAR model able to predict the activity of TRPA1 agonists with dependable accuracy was created. Considering the results obtained from different in silico studies, compound D27 has the most promising predicted properties to be a potential novel TRPA1 agonist, regarding its activity, ADMET profile, interaction, and stability in complex with TRPA1 channel. Nevertheless, further studies are necessary in order to synthetize D27 and other improved TRPA1 agonists and to evaluate their activities in experimental in vitro conditions.

## Figures and Tables

**Table 1:** Experimental and predicted activities of the compounds in the dataset with calculated validation parameters.

**Training Set**					
**Compounds**	**Experimental pEC50**	**Predicted pEC50**	**Compounds**	**Experimental pEC50**	**Predicted pEC50**
L1	4.77	4.26	L12	4.52	4.59
L2	5.05	4.87	L13	4.07	4.01
L3	5.00	5.30	L14	3.30	3.61
L4	4.02	4.01	L15	4.71	4.69
L5	3.77	3.99	L16	3.83	3.91
L6	4.22	4.10	L17	4.52	4.56
L7	4.93	4.86	L18	4.78	4.95
L8	4.00	3.90	L19	5.00	4.82
L9	5.06	5.08	L20	4.12	4.36
L10	4.92	4.60	R^2^ = 0.83		
L11	4.52	4.67	Q^2^ = 0.59		
**Test Set**					
**Compounds**	**Experimental pEC50**	**PredictedpEC50**	**Compounds**	**Experimental pEC50**	**Predicted pEC50**
L21	5.01	4.90	L27	4.72	4.87
L22	4.70	4.55	L28	4.08	4.00
L23	4.08	4.24	L29	4.52	4.59
L24	3.83	4.09	$R_{pred}^{2}$ = 0.84226 RMSEP = 0.16145		
L25	4.77	4.56	$r_{m}^{2}$ = 0.75732 $r_{m}^{‘2}$ = 0.61483		
L26	5.00	4.82	${\bar{r}}_{m}^{2}$ = 0.68608 ${\Delta r}_{m}^{2}$ = 0.14249		

**Table 2:** List of most important GRIND variables.

Variable	Node Pair	Distance [Å]	Impact	Description
450	N1–TIP	10.8–11.2	+	Distance between HBA and steric region on the benzene ring
397	O–TIP	8.4–8.8	−	Distance between HBD and steric region on the benzene ring
350	O–N1	8.4–8.8	+	Distance between HBD and HBA on the benzene ring
106	N1–N1	4.8–5.2	+	Distance between two HBAs on the benzene ring
115	N1–N1	8.4–8.8	+	Distance between two HBAs on the benzene ring
51	O–O	1.6–2.0	+	Distance between two HBDs on the benzene ring
60	O–O	5.2–5.6	+	Distance between two HBDs on the benzene ring
68	O–O	8.4–8.8	+	Distance between two HBDs on the benzene ring
388	O–TIP	4.8–5.2	−	Distance between HBD and the steric region on the benzene ring
406	O–TIP	12.0–12.4	−	Distance between HBD and the steric region on the benzene ring

**Table 3:** Calculated ADMET properties of carvacrol and selected designed compounds using ADMET Predictor (TM) version 11.0, 64-bit edition (accessed by cloud).

Compound	LogBBB	S+PrUnbnd	S+Pgp	RuleOf5	CYP_Risk	CYP_Code	TOX_MUT_Risk	TOX_MUT_Code	TOX_Risk	TOX_Code	ADMET_Risk	ADMET_Code	SynthDiff
Carvacrol	0.26	18.3	No	0	2.7	1A2; 2C9; CL	0	/	0	/	2.7	1A2; 2C9; CL	1.49
D9	−0.45	32.2	Yes	0	0	/	0.6	m_97	1	HEPX	1	HEPX	1.86
D16	−0.22	50.0	Yes	0	1	CL	0.6	S_97	1	HEPX	2	HEPX; CL	2.09
D27	−0.66	70.4	Yes	0	0	/	0	/	0	/	0	/	1.82
D82	0.16	7.9	No	0	1	CL	0.6	m_97	0	/	1	CL	1.39
D83	0.37	4.4	No	0	1	CL	0.6	m_97	0	/	2.8	Kow; fu; CL	1.52
D84	0.52	3.5	No	0	1	CL	0.6	m_97	0	/	4	Kow; Sw; fu; CL	1.97
D88	0.14	8.9	Yes	0	1	CL	0.6	NIHS	1	HEPX	2	HEPX; CL	3.92
D92	−0.26	25.9	No	0	0.3	CL	1.2	m_97; NIHS	3	Xm; HEPX; MUT	3.3	Xm; HEPX; MUT; CL	2.41
D94	0.22	13.7	No	0	1	CL	0.6	m_97	1	HEPX	2	HEPX; CL	2.46
D98	0.22	15.9	Yes	0	1.7	2C19; CL	0.6	m_97	1	HEPX	2.7	HEPX; 2C19; CL	2.60

LogBBB (logarithm of the Brain/Blood partition coefficient), S+PrUnbnd (percent UNBOUND to blood plasma proteins), S+Pgp (P-glycoprotein substrate), RuleOf5 (Lipinski’s Rule of 5), CYP_Risk (risk connected with P450 oxidation: a score in the 0–6 range), CL (high microsomal clearance), 1A2 (high 1A2 clearance), 2C19 (high 2C19 clearance), 2C9 (high 2C9 clearance), TOX_MUT_Risk (risk of mutagenicity: a score in the 0–5.4 range), m_97 and S_97 (risk of positive Ames test results with (m*) or without (S*) microsomal activation for Salmonella typhimurium strains TA97), NIHS (panel predictions are not split out with respect to S9 activation or lack thereof), TOX_Risk (risk connected with predicted toxicity: a score in the 0–6 range, Xm (carcinogenicity in mice), HEPX (hepatotoxicity), MUT (Ames positive), ADMET_Risk (Full ADMET Risk: a score in the 0–22 range) fu (fraction unbound), Kow (lipophilicity), SynthDiff (score in the 0–10 range).

**Table 4:** Virtual docking and QSAR activity results of carvacrol and selected designed TRPA1 agonists. * In the interacting residue column, residues engaged in hydrogen bonding are denoted in bold. ^§^ Carvacrol’s EC50 value is experimental.

Compound	Predicte dpEC_50_	Bind Energy [kcal/mol]	Interacting Residue *
Carvacrol	4.770 ^§^	−5.2	Ala (B:939); Leu (B:936); Leu (B:891); Ala (A:836); **Ile (A:837)**
D9	5.129	−5.0	Leu (B:936); **Ile (A:837)**
D16	5.101	−5.4	**Ser (B:943)**; Ala (B:939); Phe (A:841); **Gln (B:940)**
D27	4.996	−6.0	Ala (A:836); Leu (B:891); Ile (A:837); Leu (B:936); Phe (A:841); **Gln (B:940)**
D82	5.072	−6.3	Ser (B:943); Ile (A:837); Leu (B:936); Tyr (A:840); Phe (A:841)
D83	5.235	−6.0	**Ser (B:943)**; Phe (A:841); **Gln (B:940)**; Phe (B:947); Met (A:844); Leu (A:807); Ile (A:803); Ala (B:939)
D84	5.116	−5.9	Phe (A:841)
D88	4.999	−5.7	Phe (A:841); Ile (A:837); **Leu (B:936)**; **Ala (A:836)**; Tyr (A:840); Ala (B:939)
D92	5.087	−5.8	**Ala (B:939)**; Leu (B:936); Ile (A:837); Ser (B:887)
D94	5.049	−5.4	Ile (A:837); **Leu (B:936)**; Phe (A:841); Tyr (A:840)
D98	5.122	−5.2	Ile (A:837); **Leu (B:936)**

## Data Availability

The data presented in this study are available on request from the corresponding author.

## Supplementary Materials

The following supporting information can be downloaded at: https://www.mdpi.com/article/10.3390/pharmaceutics16070951/s1, Figure S1: Chemical structures of TRPA1 antagonists (a) IPP and (b) A967079; Table S1: Chemical structures of compounds from training and test datasets; Table S2: Chemical structures of all designed compounds with their predicted EC_50_ values.
