# Supplementary material for: Design of Novel TRPA1 Agonists Based on Structure of Natural Vasodilator Carvacrol—In Vitro and In Silico Studies
Source: Pharmaceutics. 2024 Jul 18;16(7):951. doi: 10.3390/pharmaceutics16070951 (PMC11280049; doi:10.3390/pharmaceutics16070951)
Supplement: Supplementary file 1 [file pharmaceutics-16-00951-s001.zip › pharmaceutics-3048257-supplementary.pdf]

a)

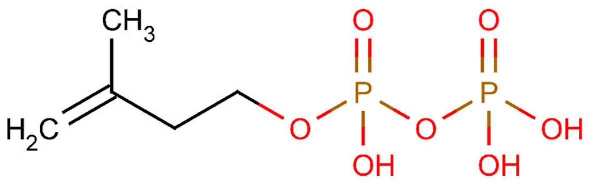

b)

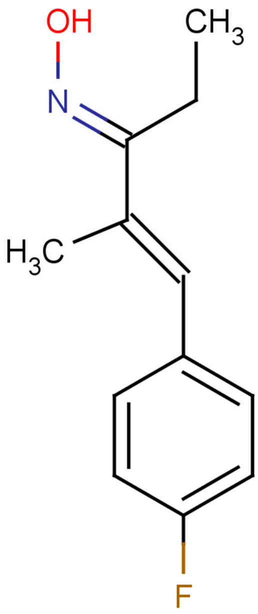

Figure S1. Chemical structures of TRPA1 antagonists a) IPP and b) A967079.

Table S1. Chemical structures of compounds from training and test datasets.

**Compounds**	**Chemical structure**
**L1**	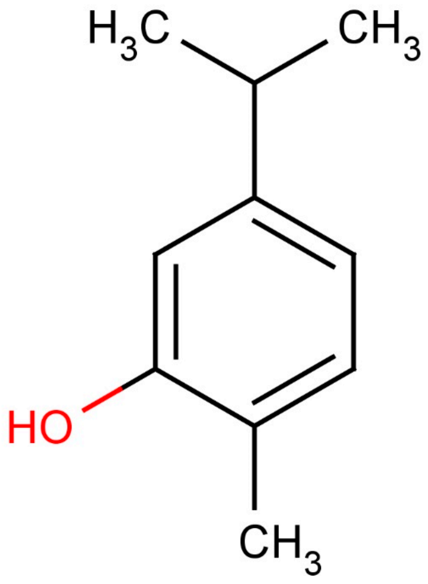

L2	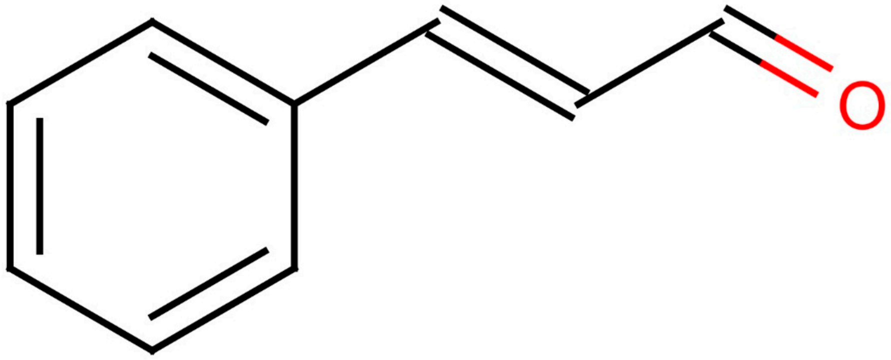
L3	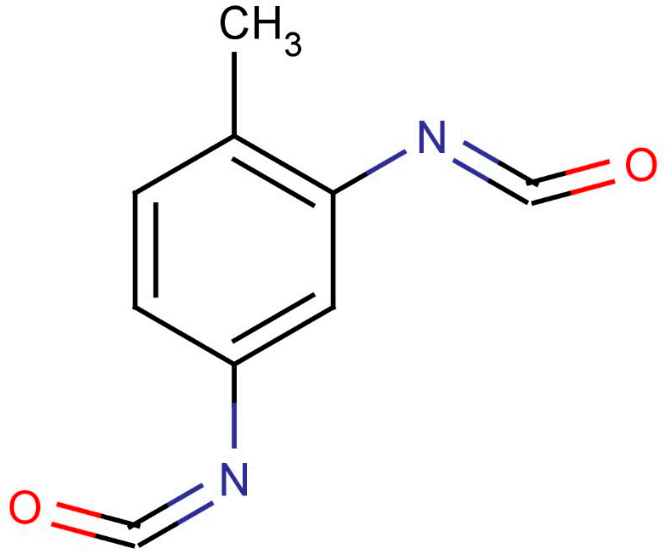

**L4**	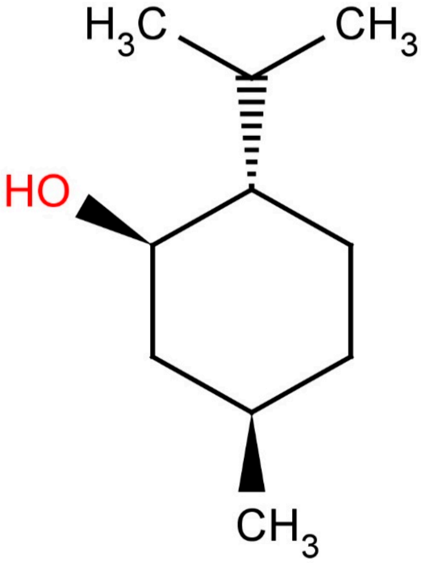
**L5**	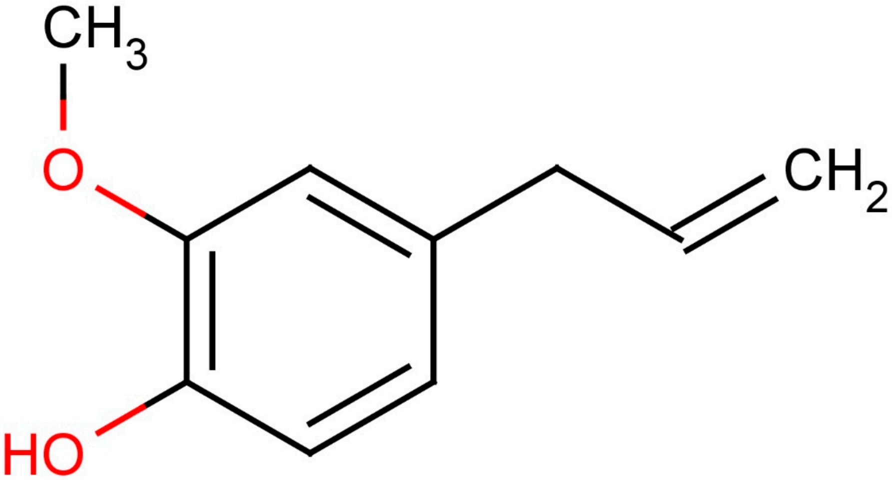

L6	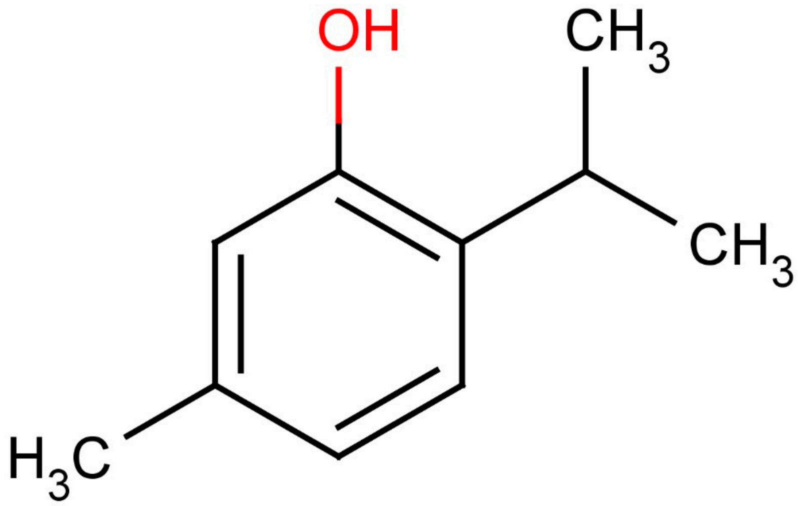
L7	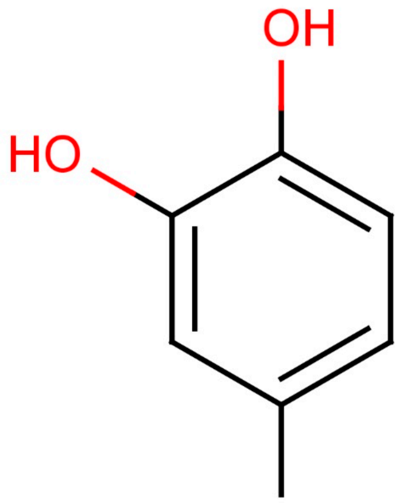

L8	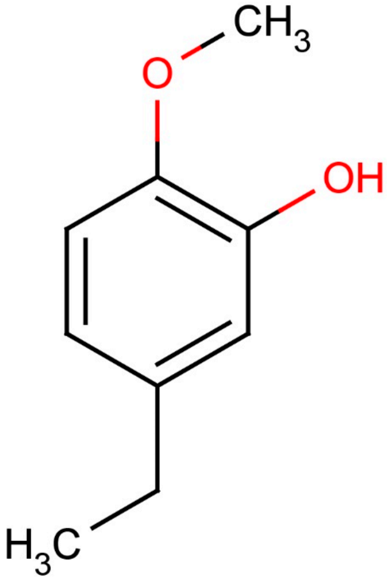
L9	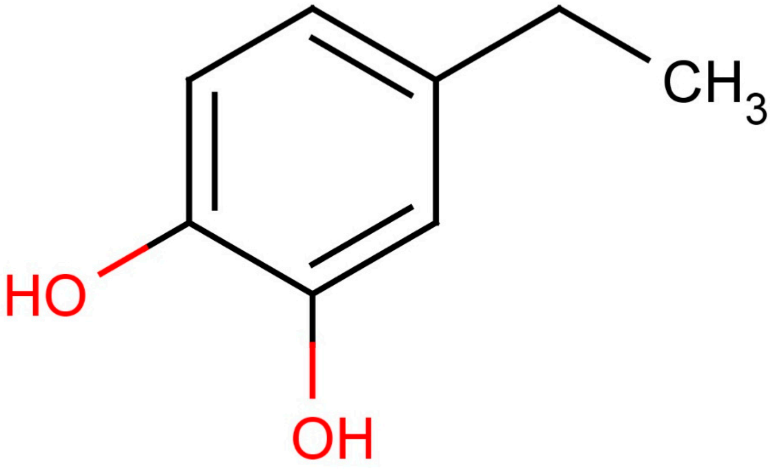

**L10**	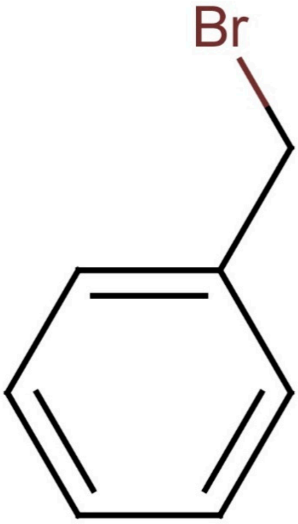
**L11**	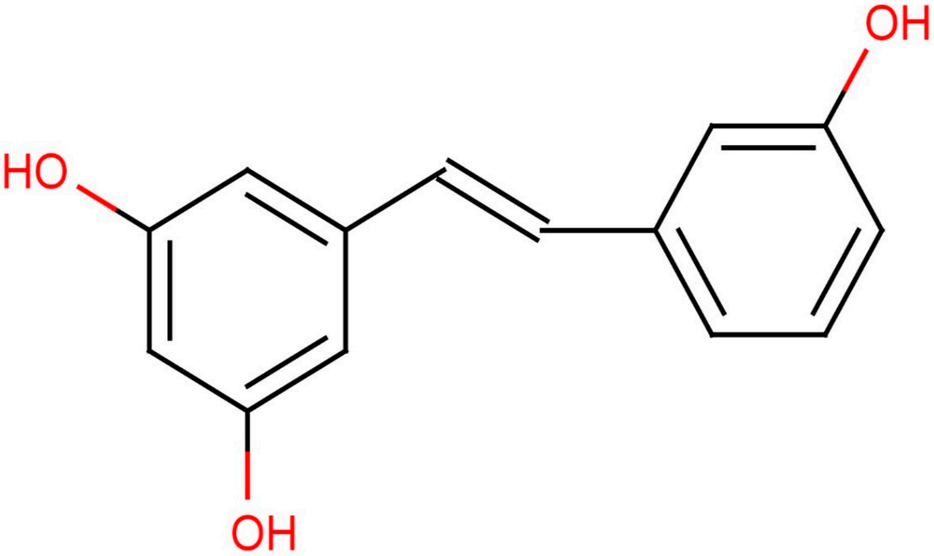

**L12**	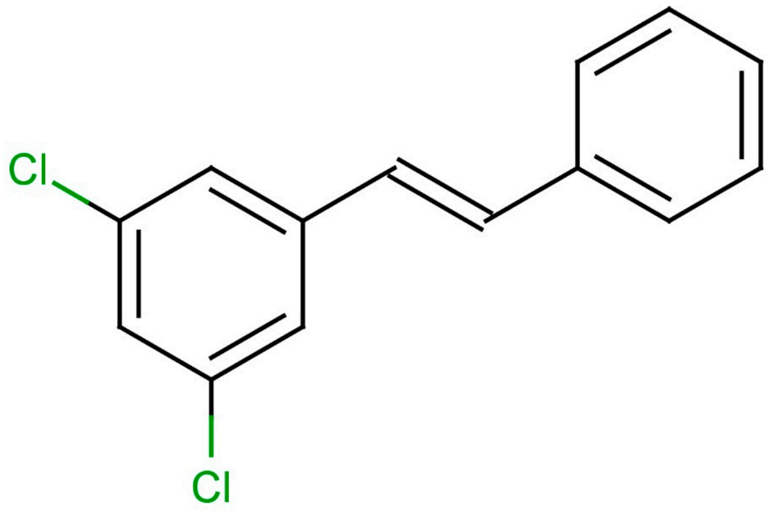
**L13**	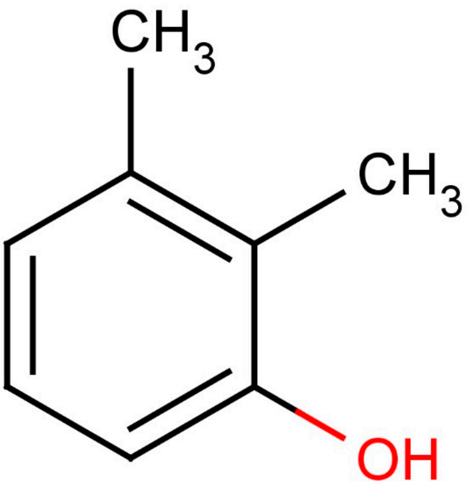

**L14**	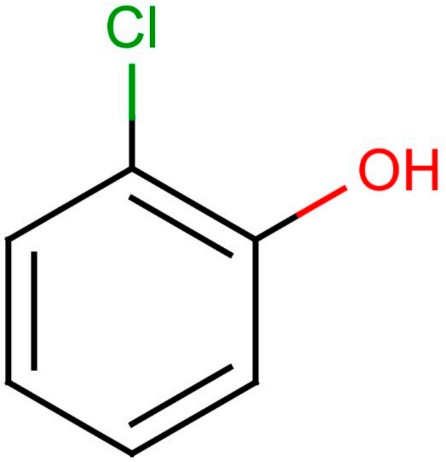
**L15**	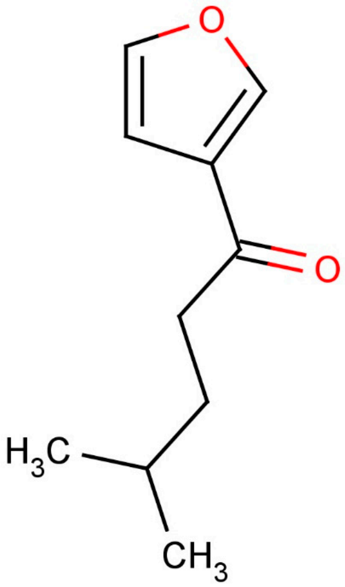

**L16**	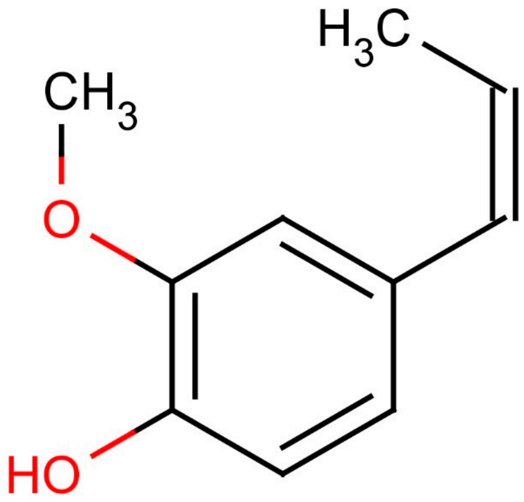
**L17**	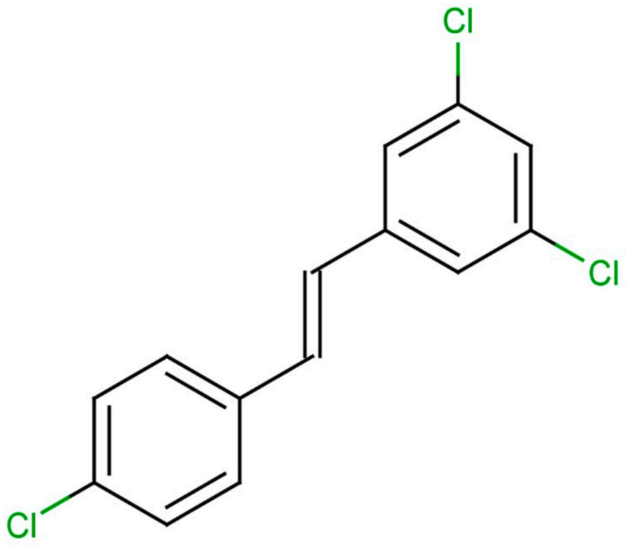

**L18**	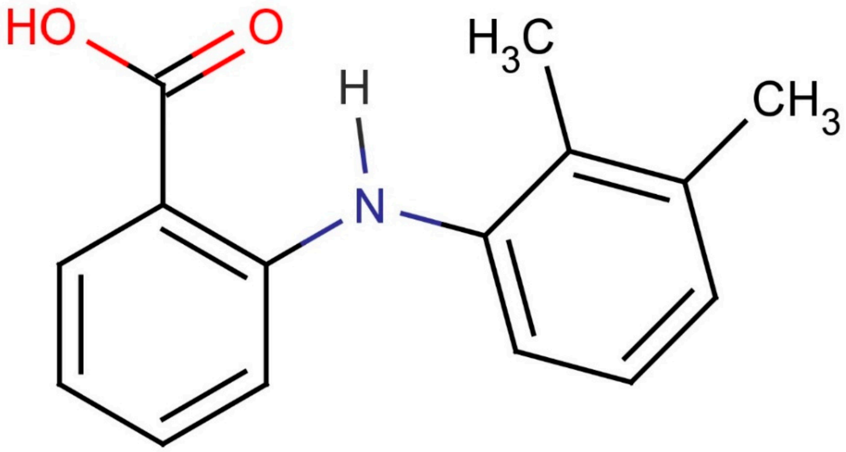
**L19**	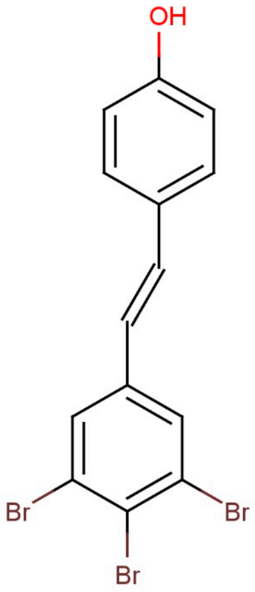
**L20**	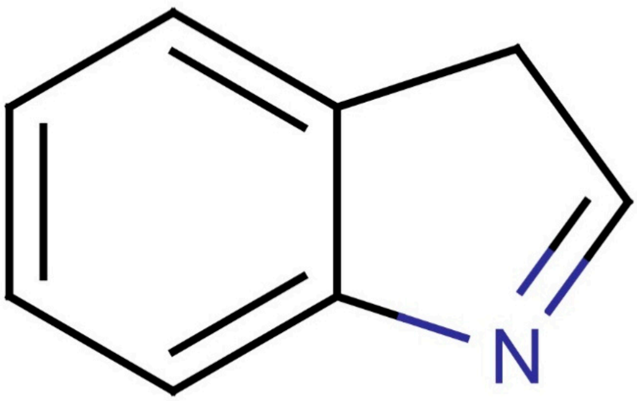

**L21**	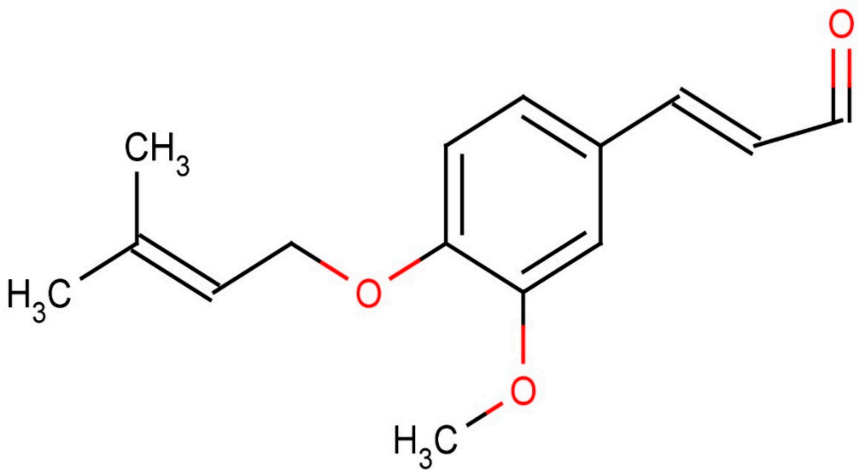
**L22**	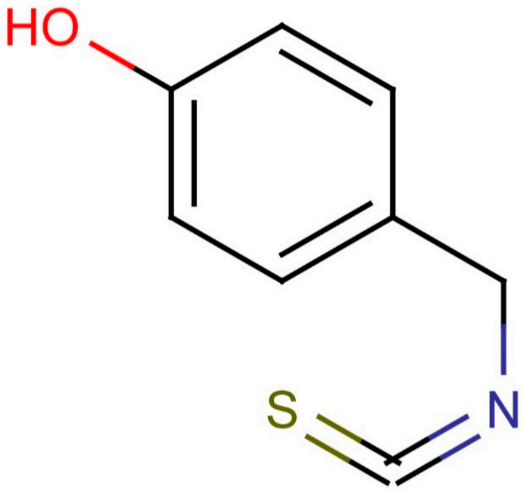

L23	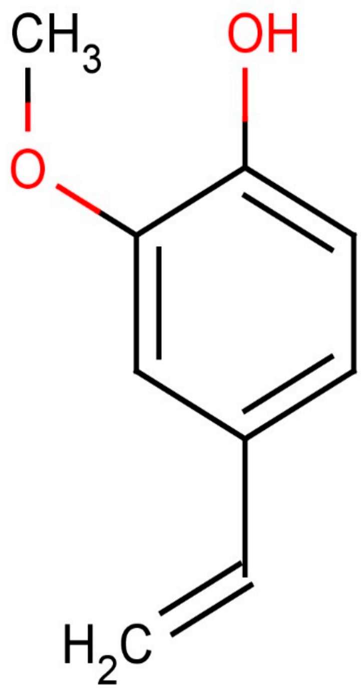
L24	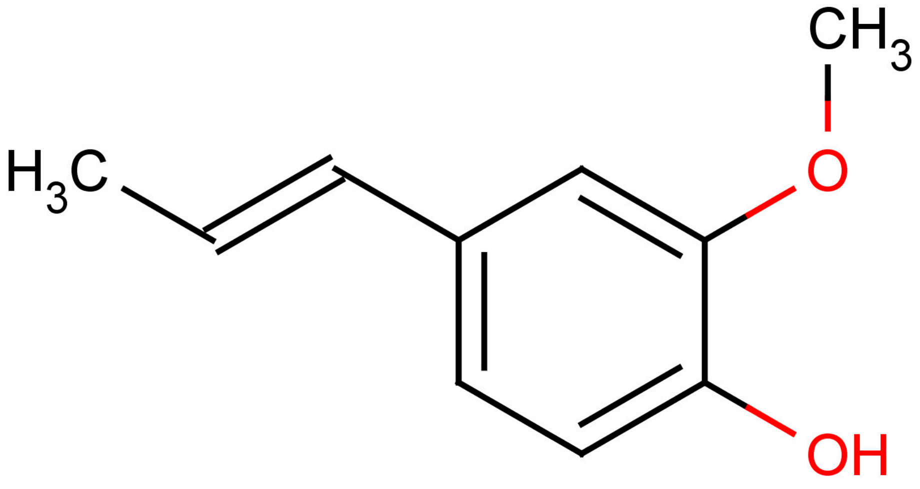

**L25**	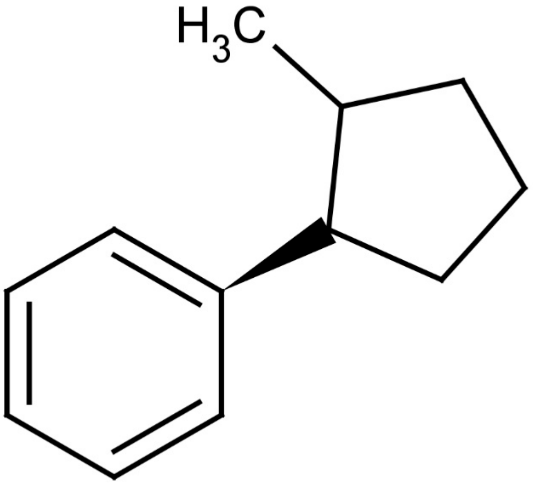
**L26**	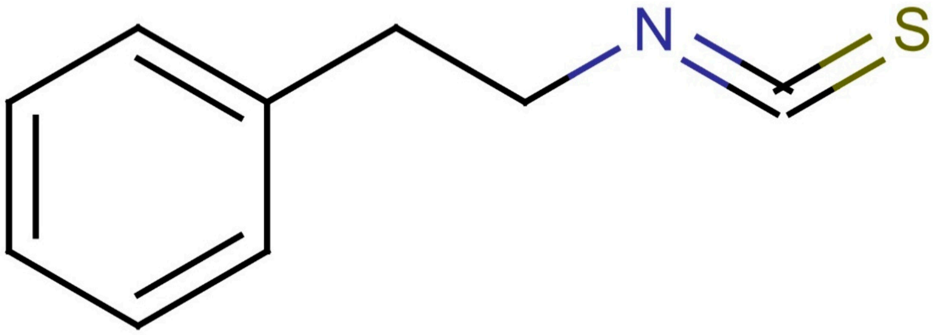
**L27**	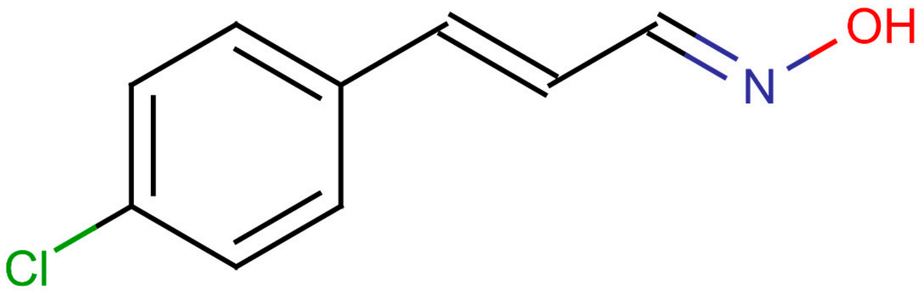

**L28**	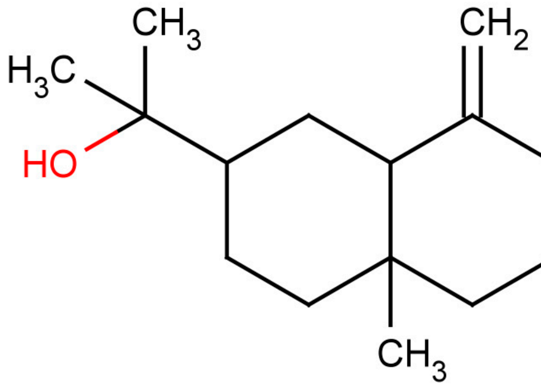
**L29**	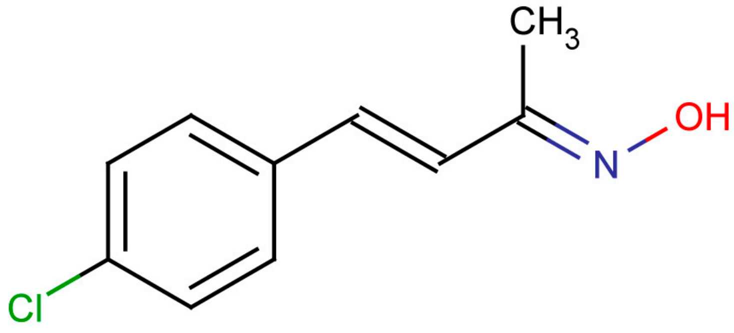

Table S2. Chemical structures of all designed compounds with their predicted EC_{50} values.

Compounds<br>Predicted EC_{50}	Chemical structure
**D1**<br>**pEC_{50} = 4.171**	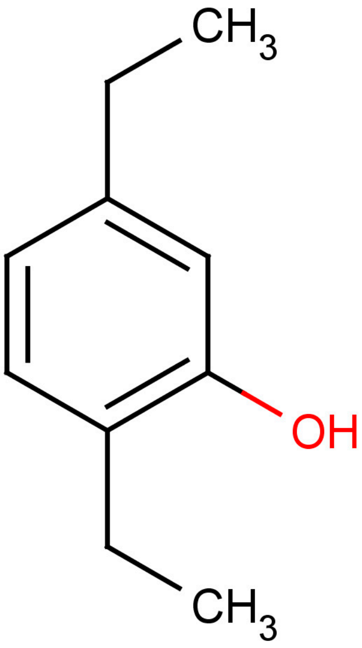
**D2**<br>**pEC_{50} = 4.015**	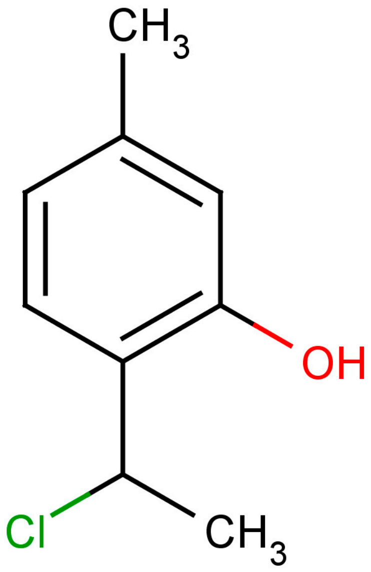

**D3**<br>**pEC$_{50}$ = 4.110**	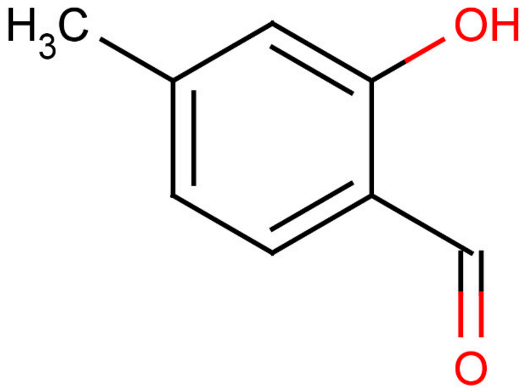
**D4**<br>**pEC$_{50}$ = 4.313**	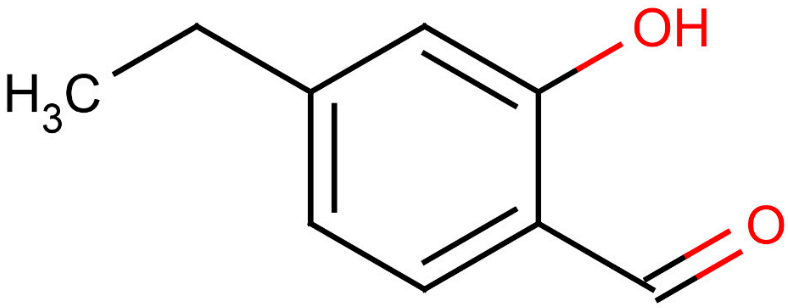
**D5**<br>**pEC$_{50}$ = 4.356**	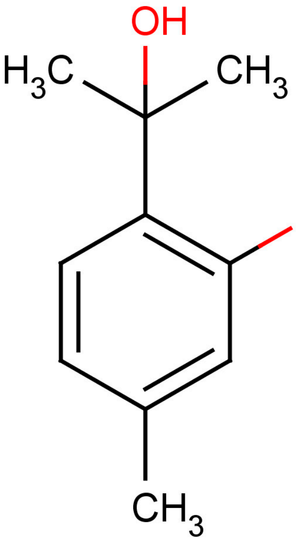

**D6**<br>**pEC_{50} = 4.741**	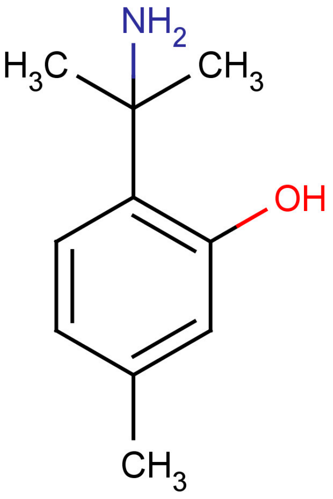
**D7**<br>**pEC_{50} = 4.206**	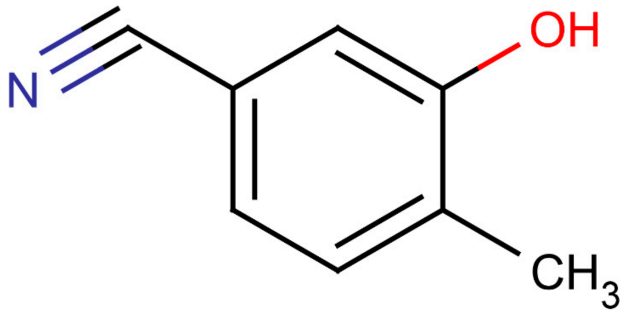
**D8**<br>**pEC_{50} = 4.251**	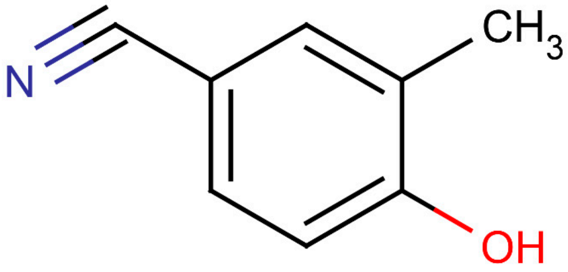

**D9**<br>**pEC$_{50}$ = 5.129**	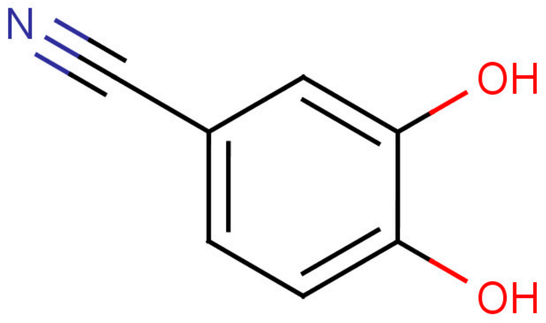
**D10**<br>**pEC$_{50}$ = 4.219**	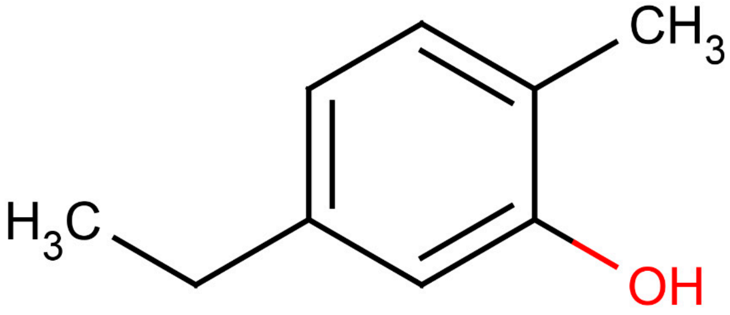
**D11**<br>**pEC$_{50}$ = 5.129**	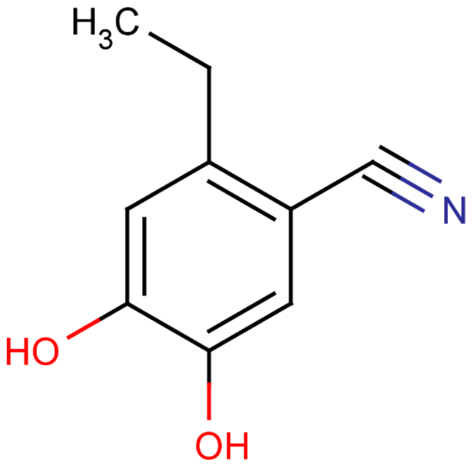

**D12**<br>**pEC$_{50}$ = 4.440**	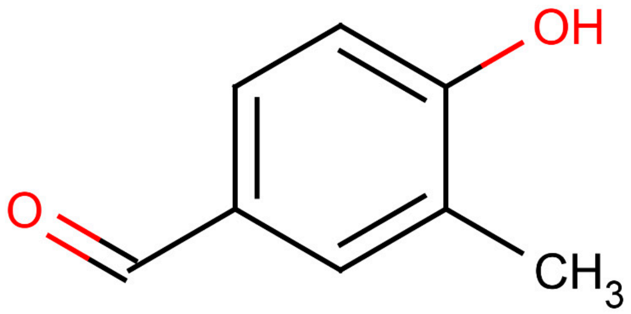
**D13**<br>**pEC$_{50}$ = 4.905**	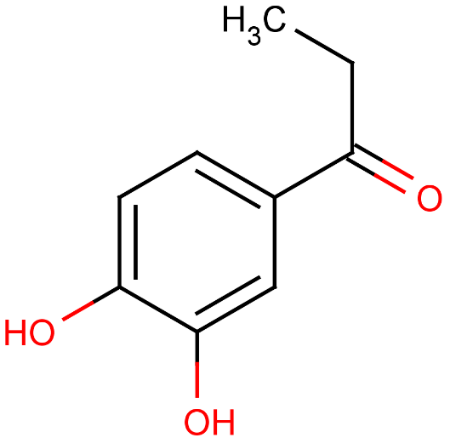
**D14**<br>**pEC$_{50}$ = 4.786**	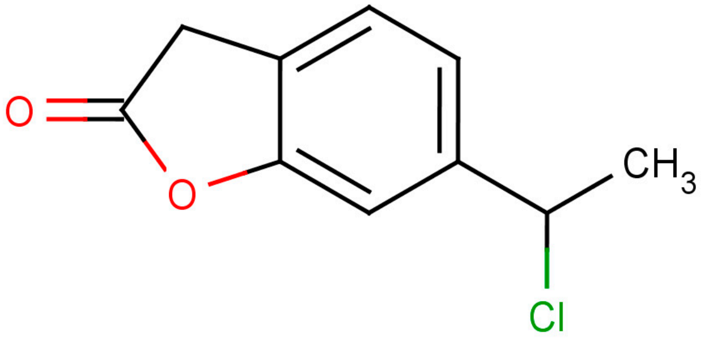

**D15**<br>pEC$_{50}$ = 4.520	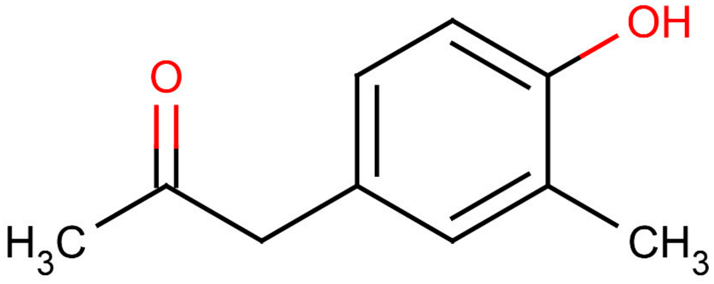
**D16**<br>pEC$_{50}$ = 5.101	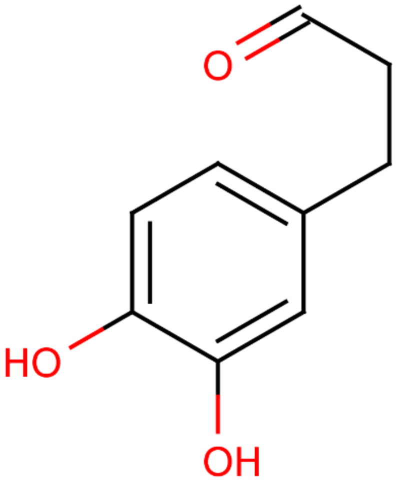
**D17**<br>pEC$_{50}$ = 4.469	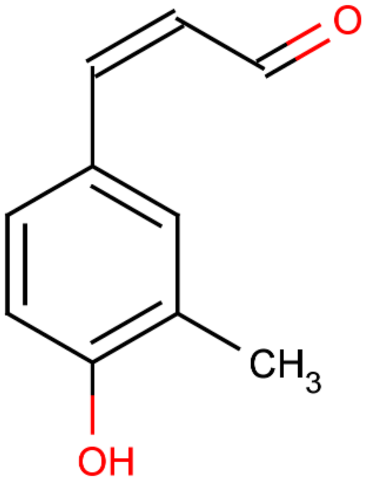

**D18**<br>**pEC50 = 4.131**	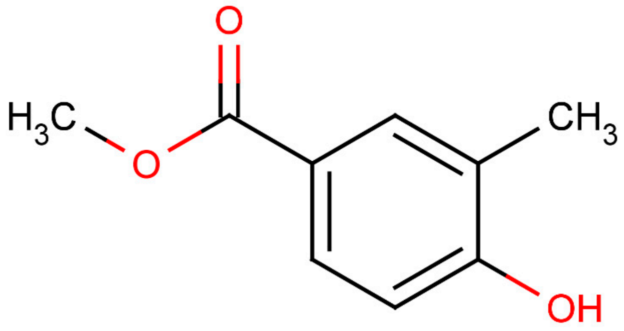
**D19**<br>**pEC50 = 4.474**	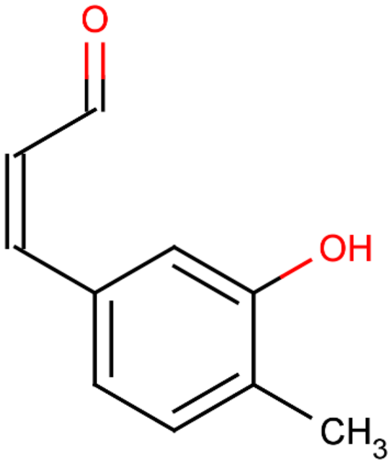
**D20**<br>**pEC50 = 4.901**	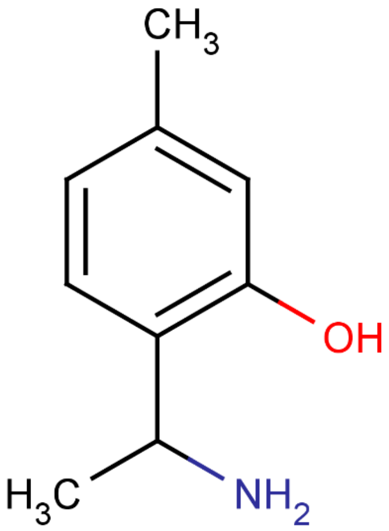

**D21** pEC$_{50}$ = 4.426	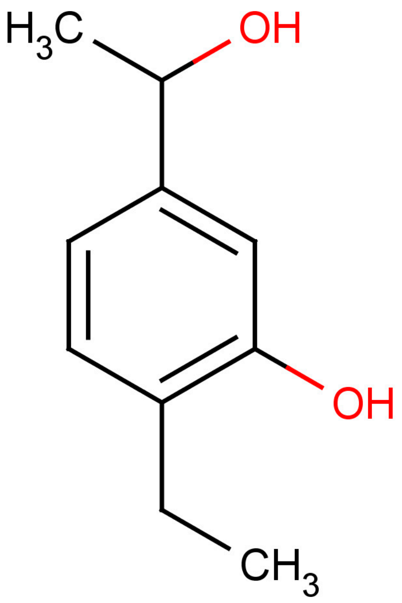
**D22** pEC$_{50}$ = 4.374	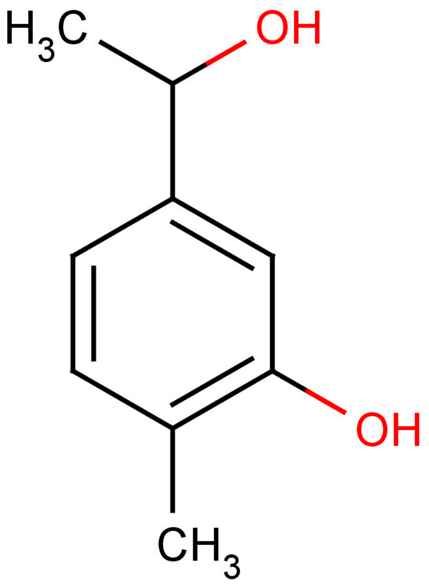

**D23**<br>**pEC$_{50}$ = 3.894**	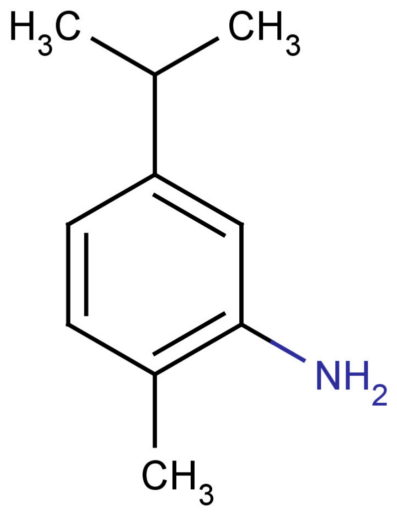
**D24**<br>**pEC$_{50}$ = 4.667**	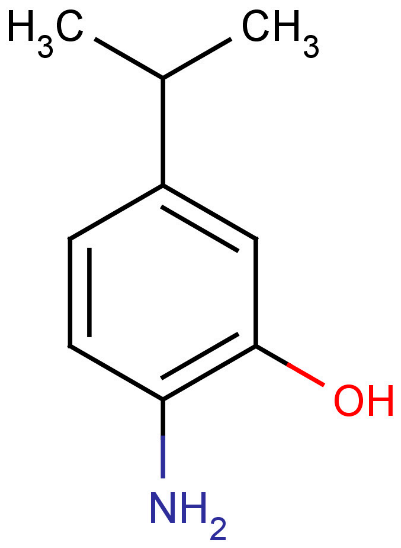

**D25**<br>**pEC$_{50}$ = 4.365**	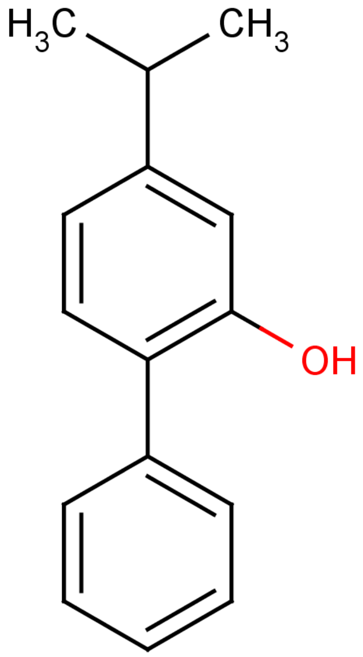
**D26**<br>**pEC$_{50}$ = 4.703**	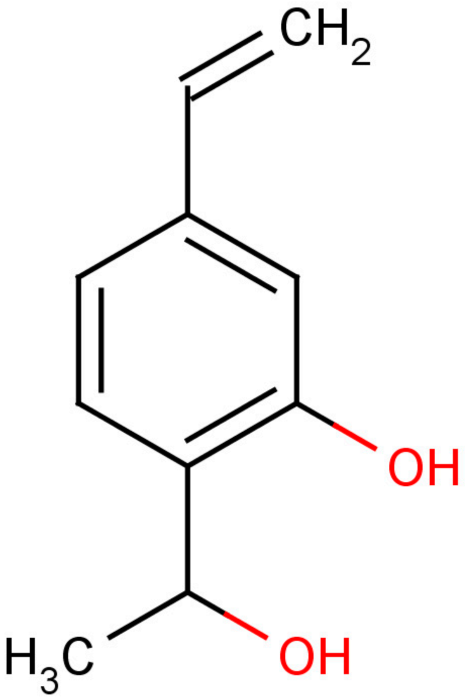

**D27**<br>**pEC_{50} = 4.996**	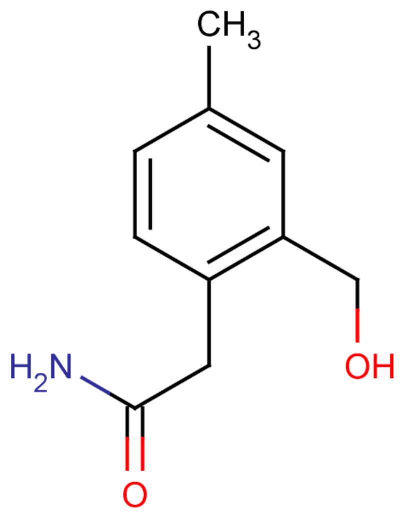
**D28**<br>**pEC_{50} = 4.486**	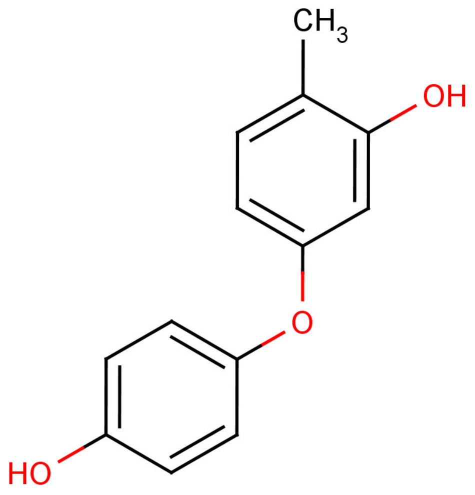

**D29**<br>**pEC_{50} = 4.740**	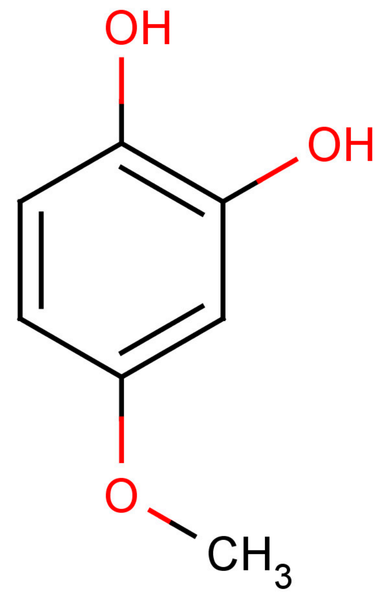
**D30**<br>**pEC_{50} = 4.718**	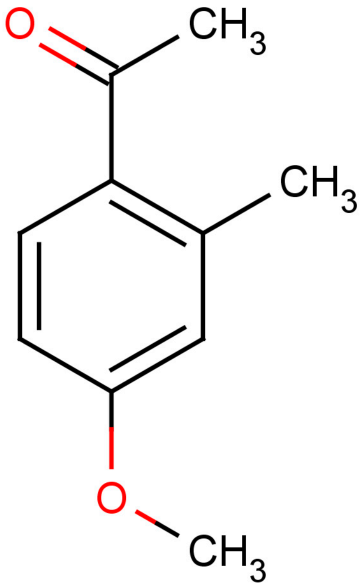

**D31**<br>pEC_{50} = 4.237	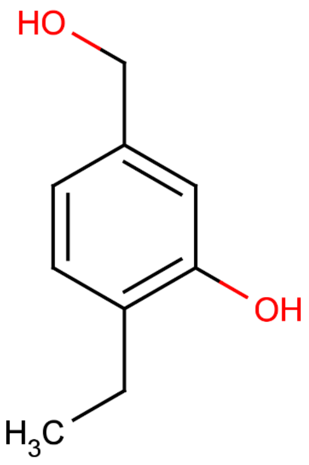
**D32**<br>pEC_{50} = 4.349	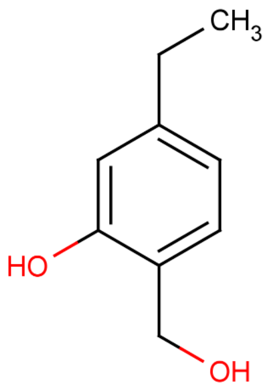
**D33**<br>pEC_{50} = 4.318	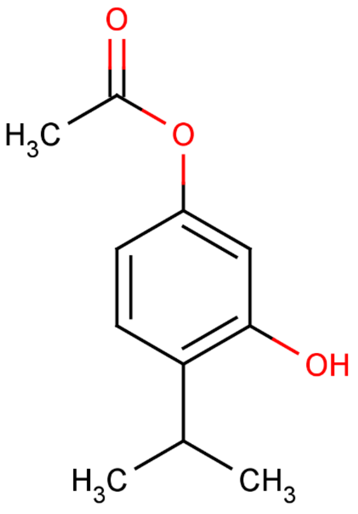

**D34**<br>**pEC$_{50}$ = 4.782**	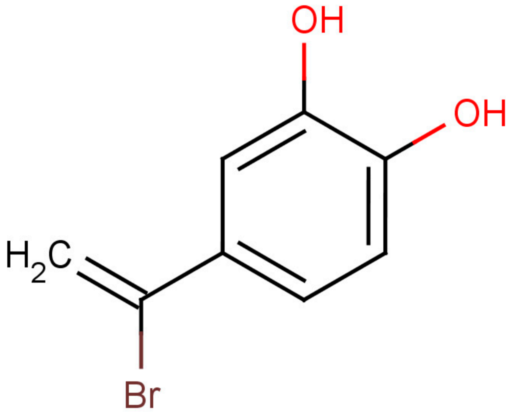
**D35**<br>**pEC$_{50}$ = 4.561**	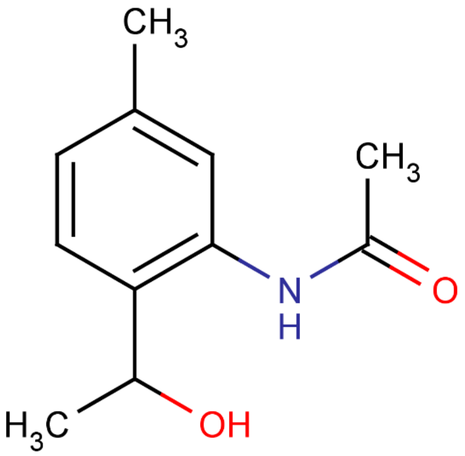
**D36**<br>**pEC$_{50}$ = 4.551**	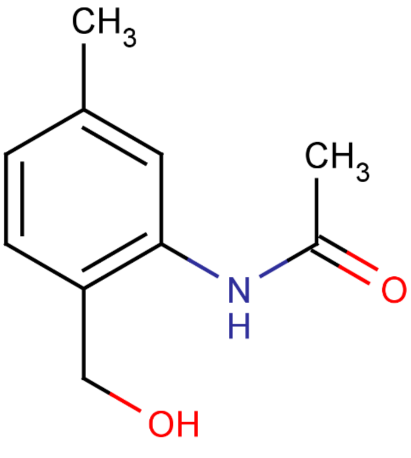

**D37**<br>**pEC$_{50}$ = 4.024**	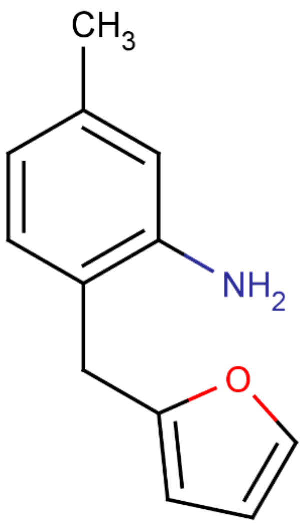
**D38**<br>**pEC$_{50}$ = 4.182**	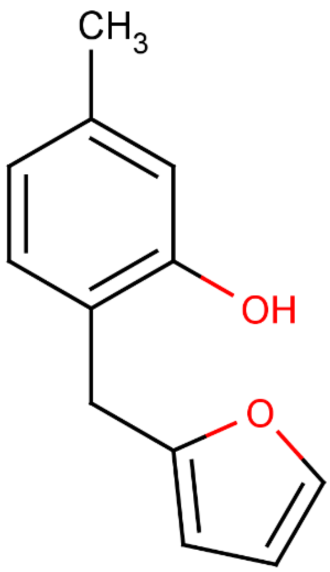

**D39**<br>**pEC50 = 4.382**	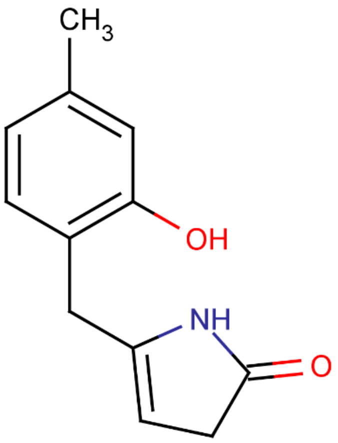
**D40**<br>**pEC50 = 4.103**	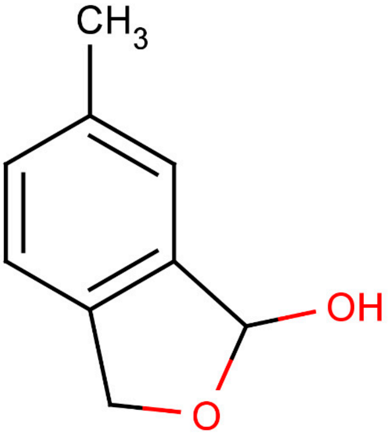
**D41**<br>**pEC50 = 4.284**	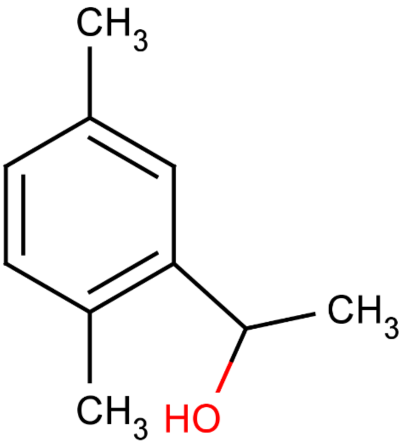

**D42**<br>**pEC_{50} = 4.444**	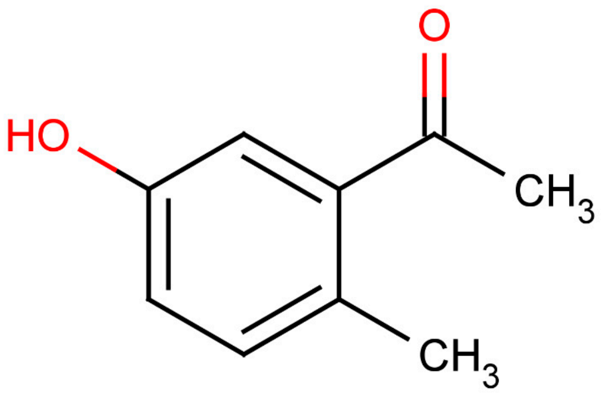
**D43**<br>**pEC_{50} = 4.311**	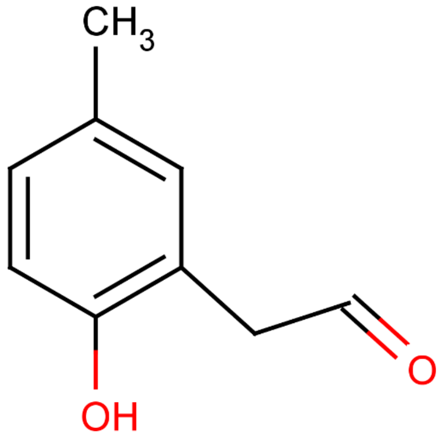
**D44**<br>**pEC_{50} = 4.317**	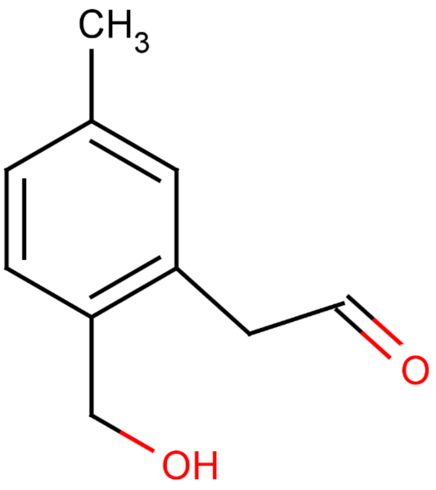

**D45**<br>**pEC50 = 4.359**	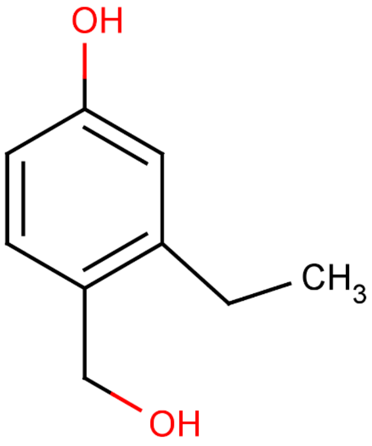
**D46**	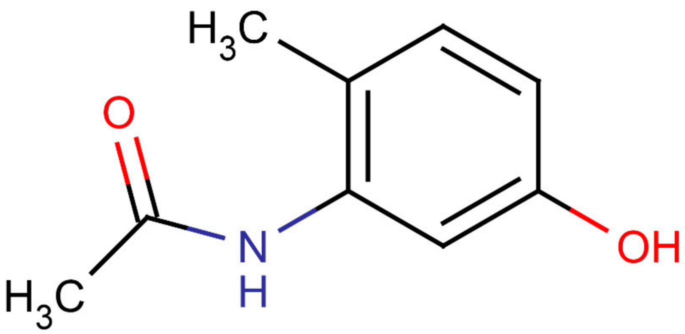
**D47**<br>**pEC50 = 4.410**	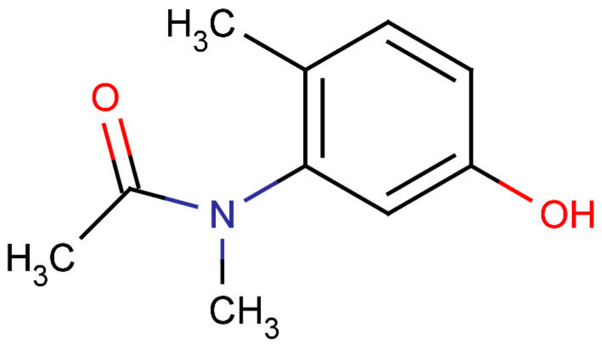

**D48** pEC$_{50}$ = 4.542	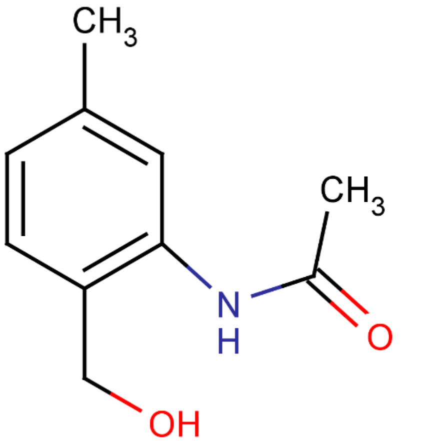
**D49** pEC$_{50}$ = 4.522	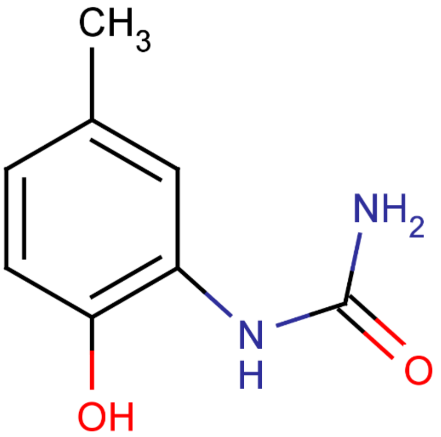
**D50** pEC$_{50}$ = 4.538	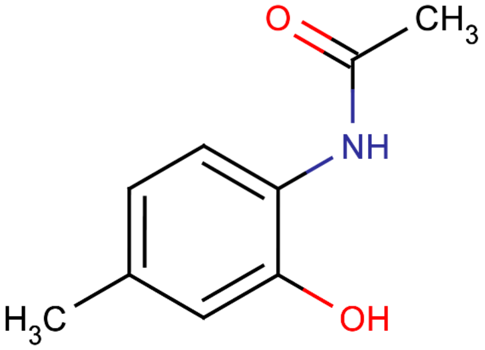

**D51**<br>**pEC$_{50}$ = 4.259**	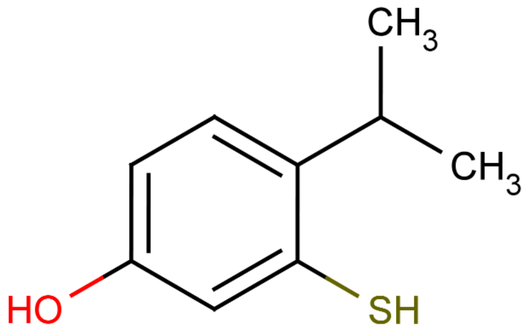
**D52**<br>**pEC$_{50}$ = 4.614**	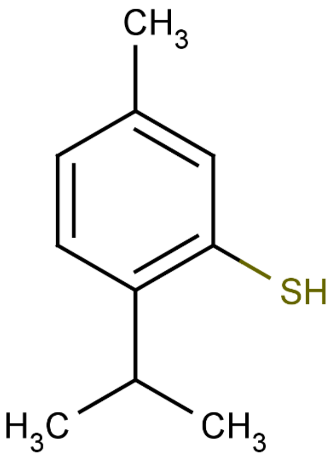
**D53**<br>**pEC$_{50}$ = 4.784**	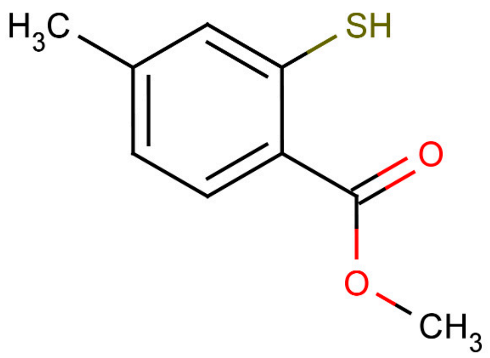

**D54**<br>pEC_{50} = 4.419	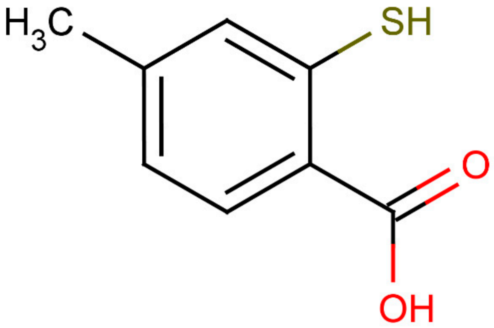
**D55**<br>pEC_{50} = 3.981	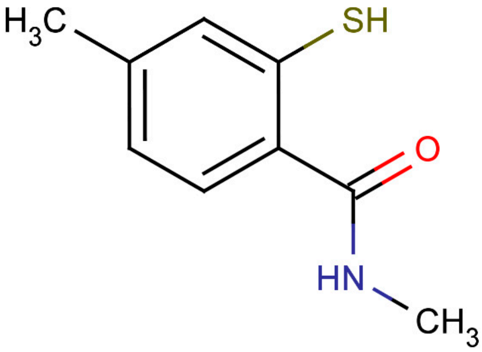
**D56**<br>pEC_{50} = 4.027	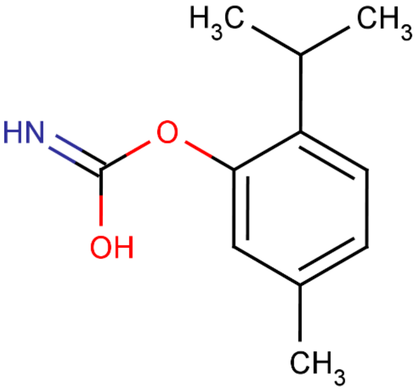

**D57**<br>**pEC$_{50}$ = 4.601**	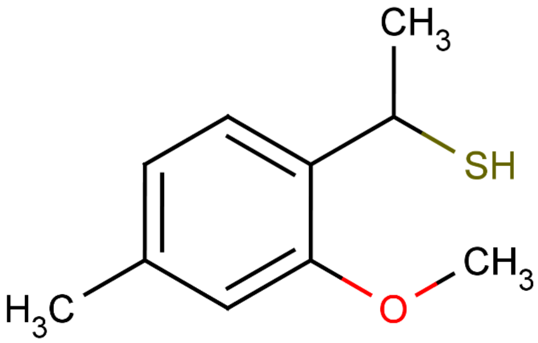
**D58**<br>**pEC$_{50}$ = 4.086**	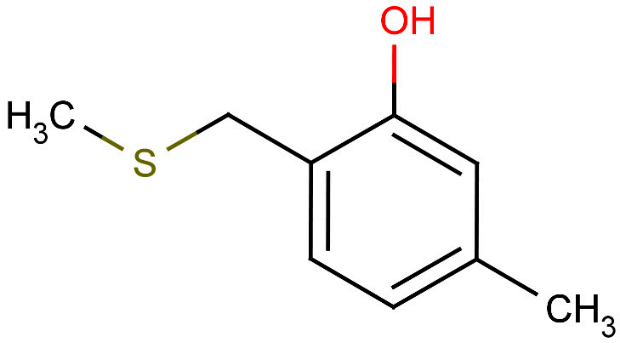
**D59**<br>**pEC$_{50}$ = 4.846**	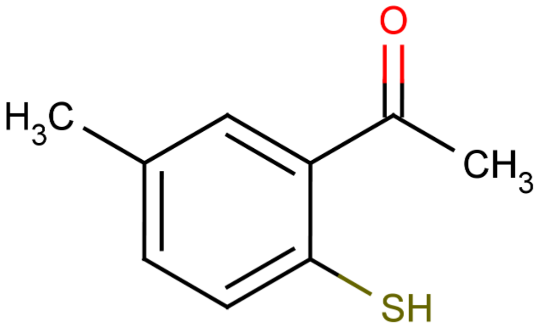
**D60**<br>**pEC$_{50}$ = 4.806**	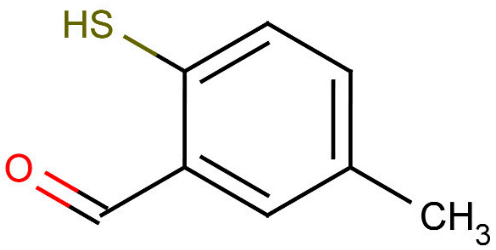

**D61**<br>**pEC$_{50}$ = 4.053**	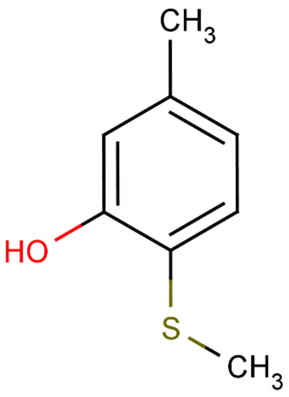
**D62**<br>**pEC$_{50}$ = 4.322**	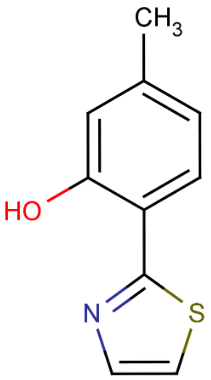
**D63**<br>**pEC$_{50}$ = 3.974**	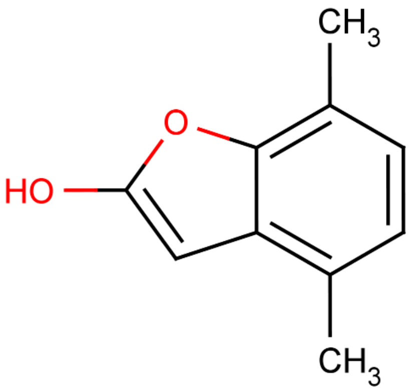

**D64**<br>**pEC_{50} = 3.904**	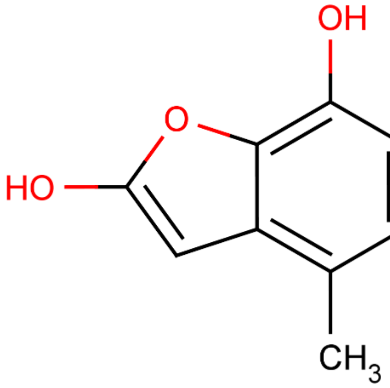
**D65**<br>**pEC_{50} = 4.235**	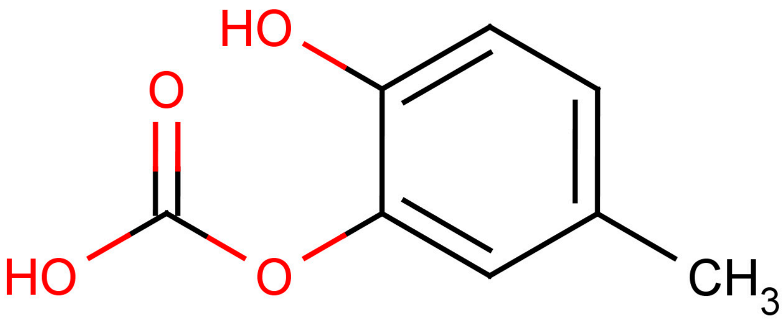
**D66**<br>**pEC_{50} = 4.546**	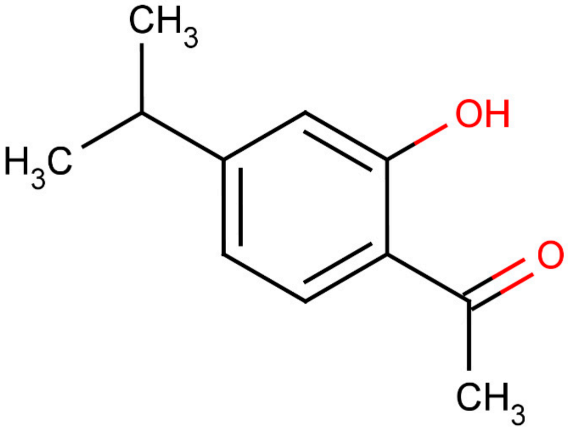

**D67**<br>**pEC$_{50}$ = 4.419**	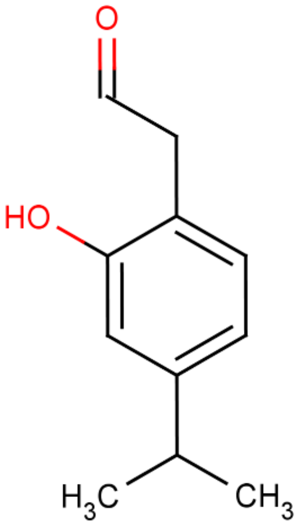
**D68**<br>**pEC$_{50}$ = 4.236**	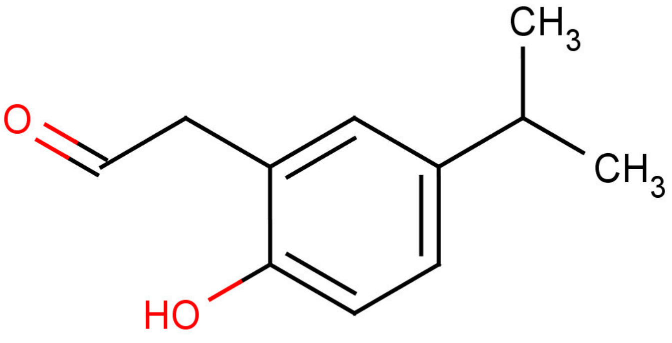
**D69**<br>**pEC$_{50}$ = 3.855**	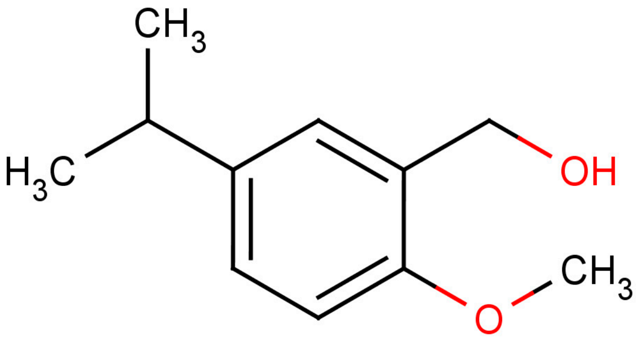

**D70**<br>**pEC$_{50}$ = 4.389**	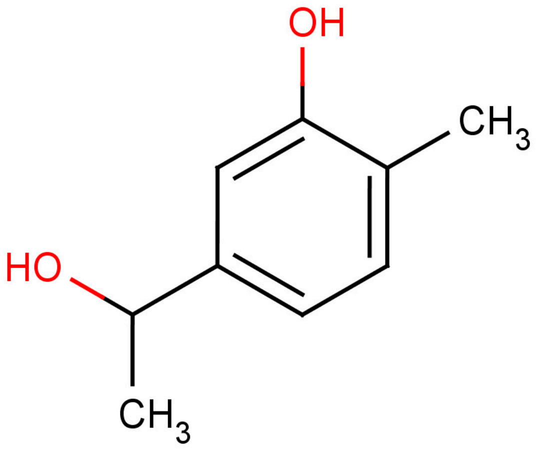
**D71**<br>**pEC$_{50}$ = 4.214**	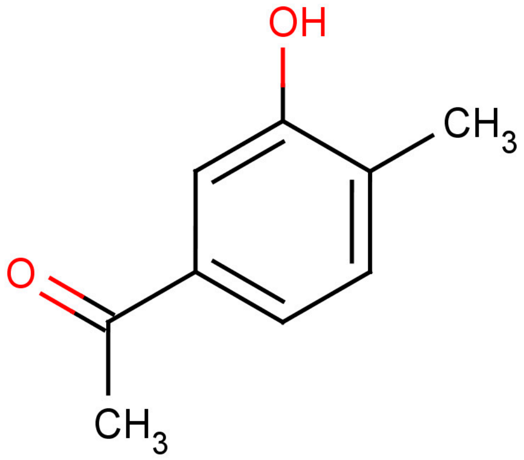

**D72**<br>$pEC_{50} = 3.903$	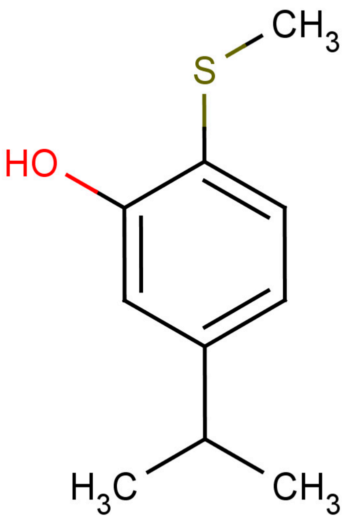
**D73**<br>$pEC_{50} = 4.162$	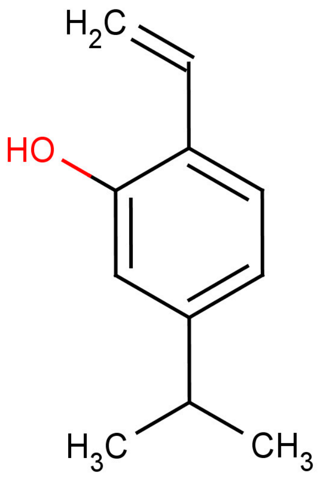
**D74**<br>$pEC_{50} = 4.804$	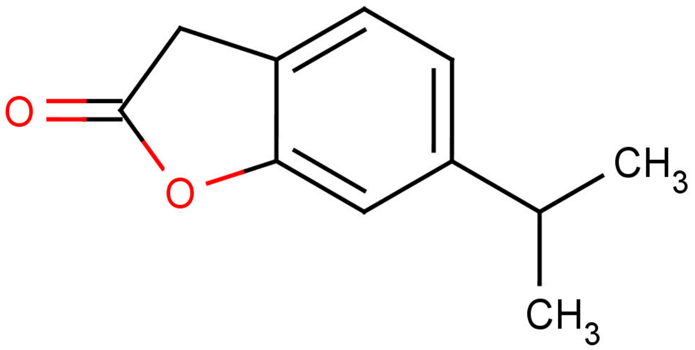

**D75**<br>pEC_{50} = 4.372	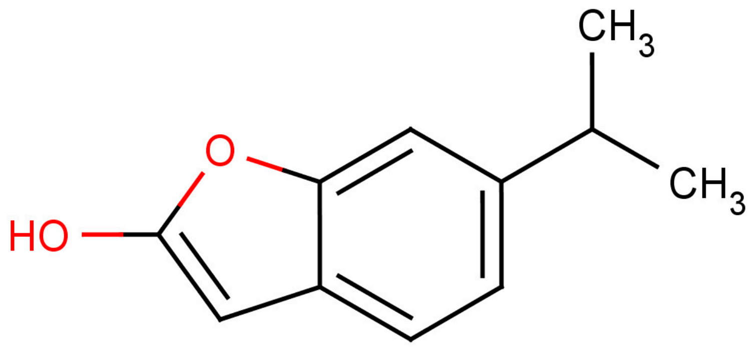
**D76**<br>pEC_{50} = 4.071	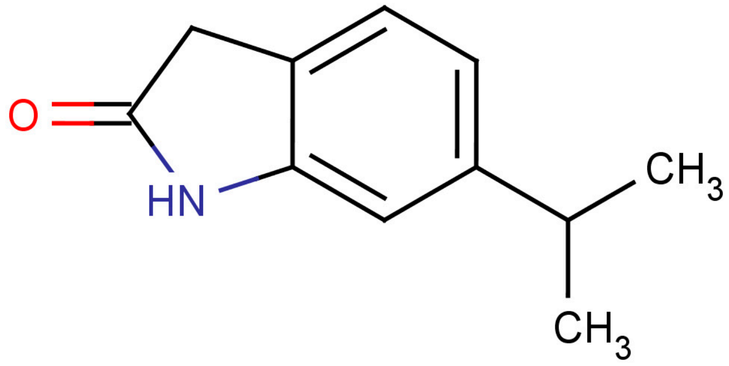
**D77**<br>pEC_{50} = 3.928	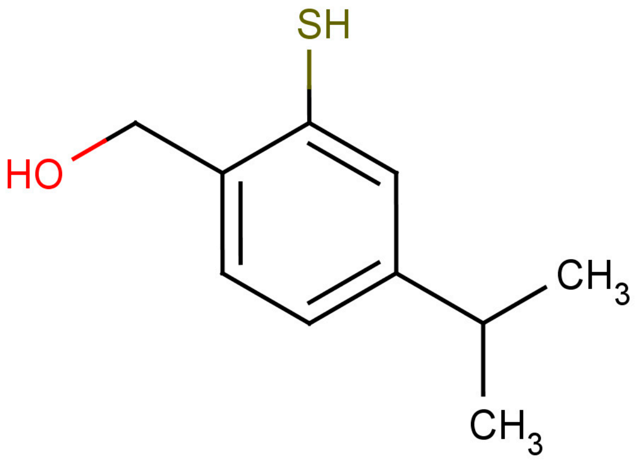

**D78**<br>**pEC_{50} = 4.220**	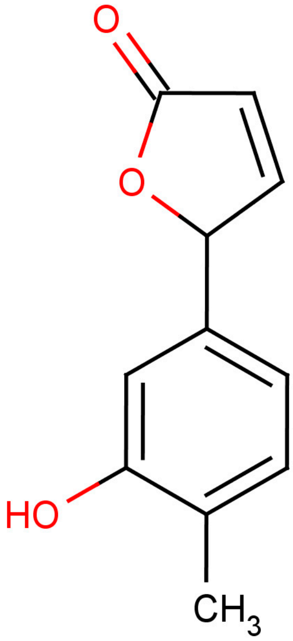
**D79**<br>**pEC_{50} = 4.548**	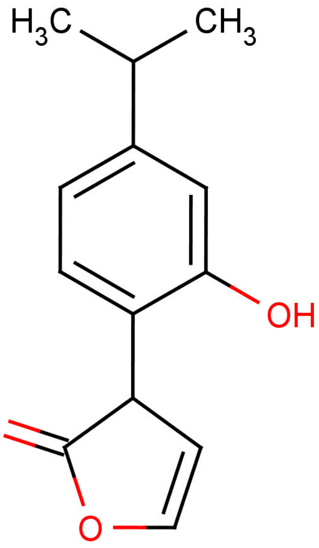

**D80**<br>**pEC$_{50}$ = 4.391**	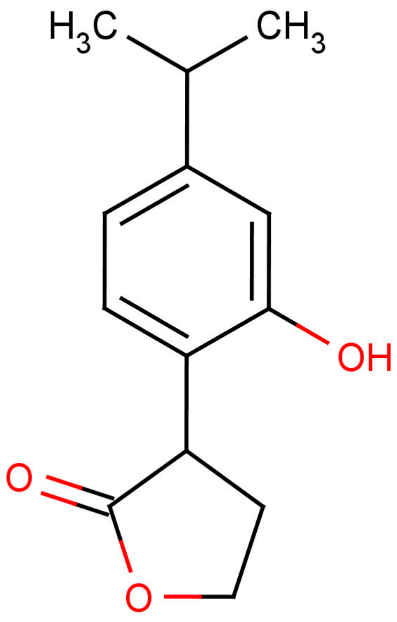
**D81**<br>**pEC$_{50}$ = 4.906**	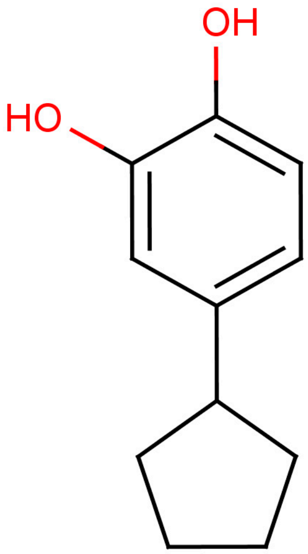

**D82**<br>$pEC_{50} = 5.072$	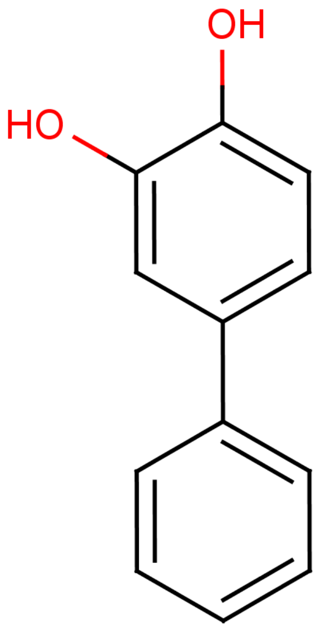
**D83**<br>$pEC_{50} = 5.235$	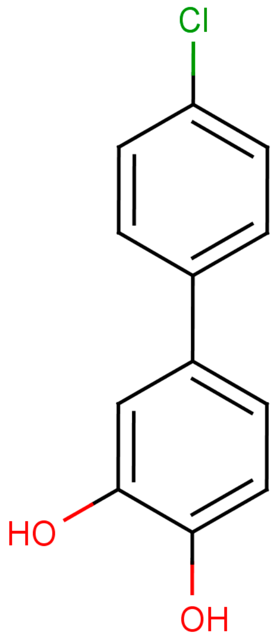

**D84** pEC$_{50}$ = 5.116	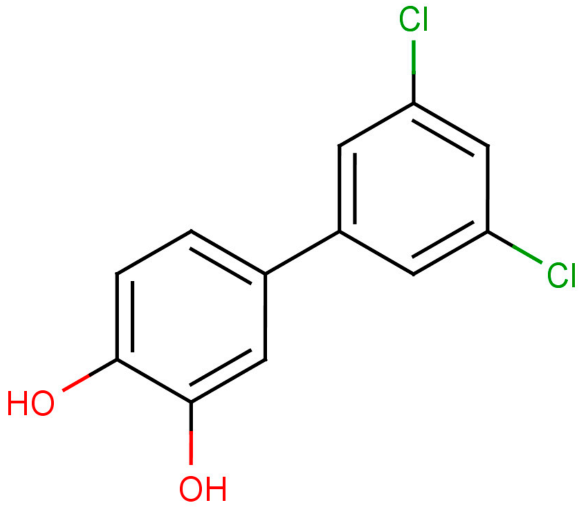
**D85** pEC$_{50}$ = 4.137	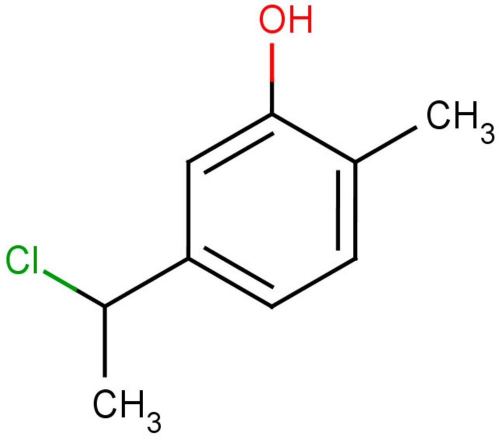

**D86**<br>**pEC50 = 4.892**	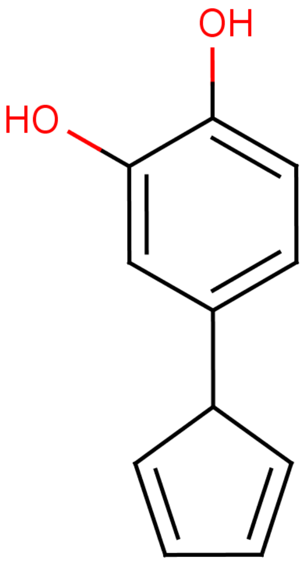
**D87**<br>**pEC50 = 4.069**	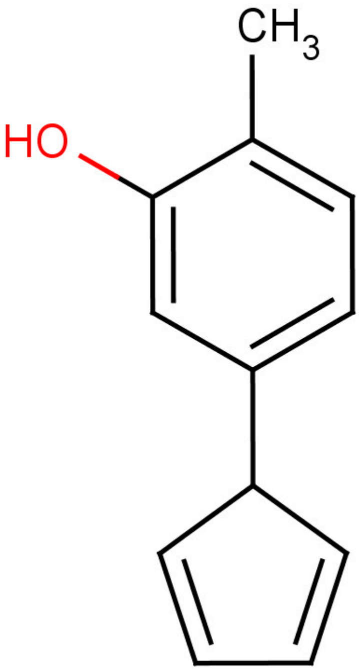

**D88**<br>**pEC_{50} = 4.999**	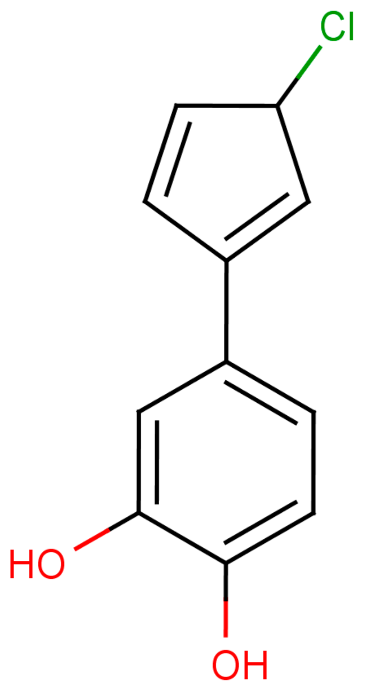
**D89**<br>**pEC_{50} = 4.900**	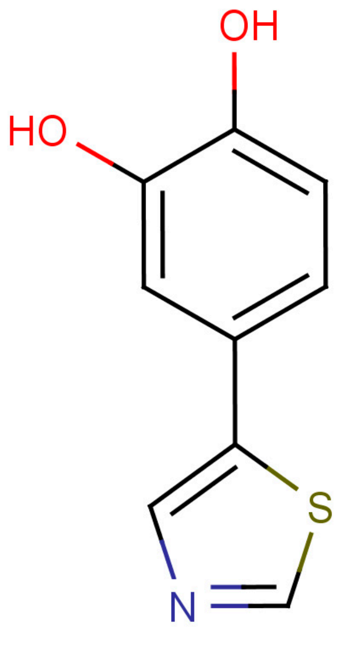

**D90**<br>**pEC$_{50}$ = 5.037**	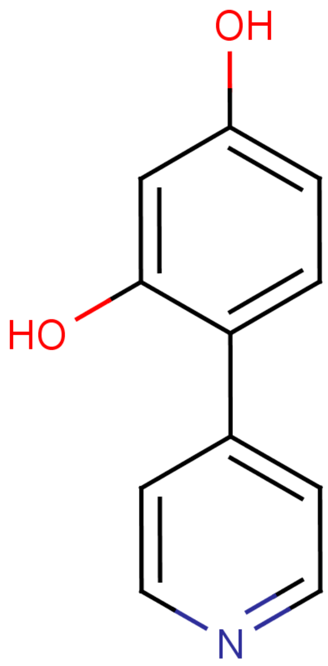
**D91**<br>**pEC$_{50}$ = 4.903**	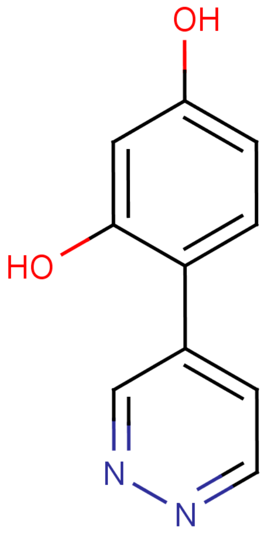

**D92**<br>**pEC_{50} = 5.087**	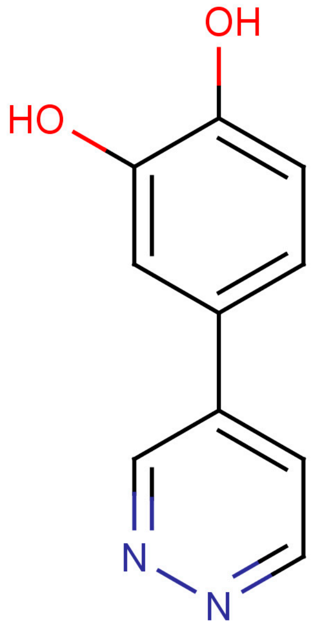
**D93**<br>**pEC_{50} = 4.838**	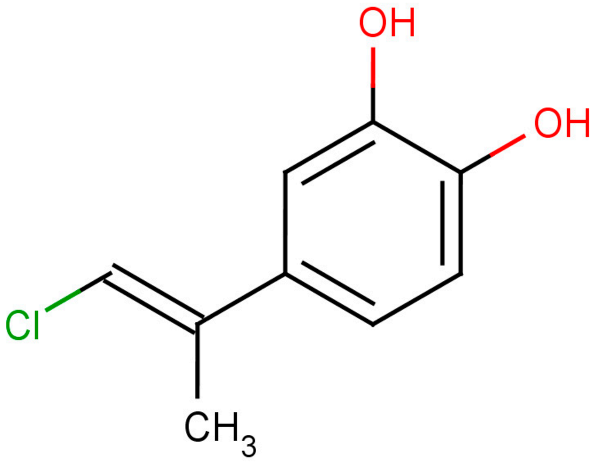

**D94**<br>**pEC_{50} = 5.049**	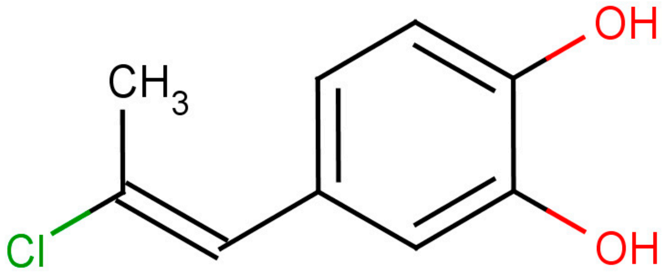
**D95**<br>**pEC_{50} = 3.763**	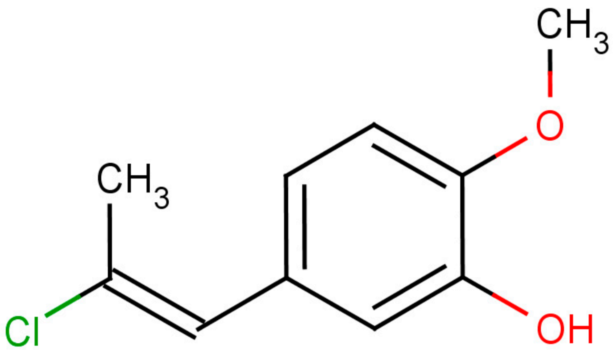
**D96**<br>**pEC_{50} = 4.669**	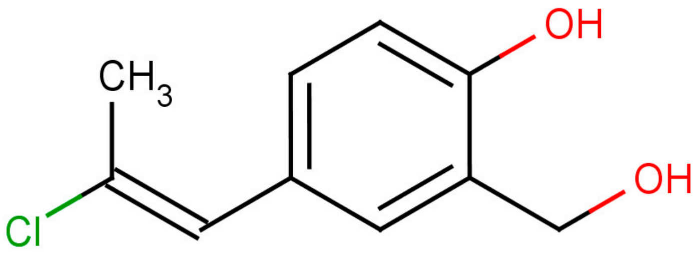

**D97**<br>**pEC$_{50}$ = 4.827**	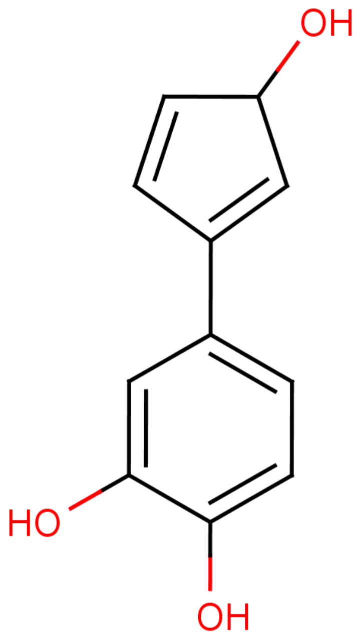
**D98**<br>**pEC$_{50}$ = 5.122**	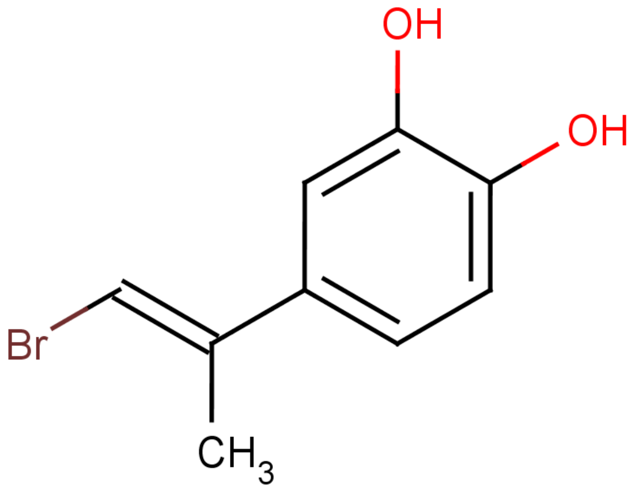

**D99**<br>**pEC_{50} = 4.863**	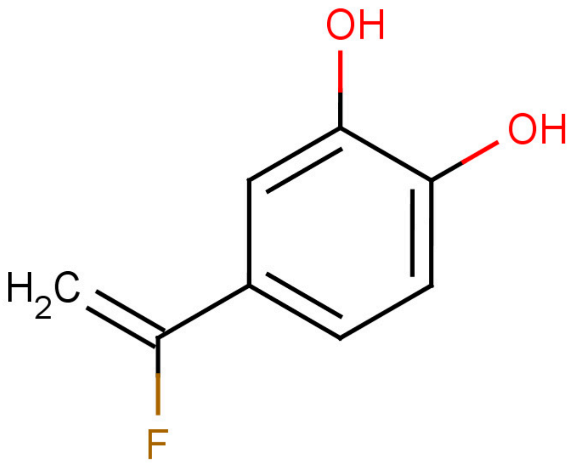
**D100**<br>**pEC_{50} = 4.805**	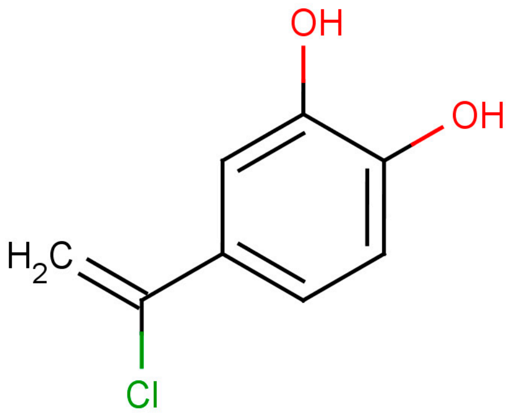
